# Coordination Thermodynamic Control of Magnetic Domain Configuration Evolution toward Low-Frequency Electromagnetic Attenuation

**DOI:** 10.1007/s40820-025-01948-1

**Published:** 2026-01-08

**Authors:** Tong Huang, Dan Wang, Xue He, Zhaobo Feng, Zhiqiang Xiong, Yuqi Luo, Yuhui Peng, Guangsheng Luo, Xuliang Nie, Mingyue Yuan, Chongbo Liu, Renchao Che

**Affiliations:** 1 Key Laboratory of Jiangxi Province for Persistent Pollutants Control and Resources Recycle, School of Environmental and Chemical Engineering, Nanchang Hangkong University, Nanchang, 330063 People’s Republic of China; 2 State Environmental Protection Key Laboratory of Environmental Risk Assessment and Control On Chemical Process, School of Mechanical and Power Engineering, East China University of Science and Technology, Shanghai, 200237 People’s Republic of China; 3 Key Laboratory of Nondestructive Testing, Ministry of Education, School of Instrument Science and Optoelectronic Engineering, Nanchang Hangkong University, Nanchang, 330063 People’s Republic of China; 4 School of Physics and Materials, Nanchang University, Nanchang, 330031 People’s Republic of China; 5 College of Chemistry & Materials, Jiangxi Agricultural University, Nanchang, 330045 People’s Republic of China; 6 Laboratory of Advanced Materials, Shanghai Key Lab of Molecular Catalysis and Innovative Materials, Academy for Engineering & Technology, Fudan University, Shanghai, 200438 People’s Republic of China

**Keywords:** Thermodynamically controlled coordination strategy, Magnetic domain configuration, Low-frequency electromagnetic wave absorption, Electrical/magnetic coupling, Multifunction

## Abstract

The precise tuning of magnetic nanoparticle size and spacing directly influences the alignment of intrinsic magnetic moments and magnetic domains, thereby shaping magnetic properties. However, the dynamic evolution mechanisms of magnetic domain configurations in relation to electromagnetic (EM) attenuation behavior remain poorly understood. To address this gap, a thermodynamically controlled periodic coordination strategy is proposed to achieve precise modulation of magnetic nanoparticle spacing. This approach unveils the evolution of magnetic domain configurations, progressing from individual to coupled and ultimately to crosslinked domain configurations. A unique magnetic coupling phenomenon surpasses the Snoek limit in low-frequency range, which is observed through micromagnetic simulation. The crosslinked magnetic configuration achieves effective low-frequency EM wave absorption at 3.68 GHz, encompassing nearly the entire C-band. This exceptional magnetic interaction significantly enhances radar camouflage and thermal insulation properties. Additionally, a robust gradient metamaterial design extends coverage across the full band (2–40 GHz), effectively mitigating the impact of EM pollution on human health and environment. This comprehensive study elucidates the evolution mechanisms of magnetic domain configurations, addresses gaps in dynamic magnetic modulation, and provides novel insights for the development of high-performance, low-frequency EM wave absorption materials.

**Supplementary Information:**

The online version contains supplementary material available at 10.1007/s40820-025-01948-1.

## Highlights

- A periodic coordination thermodynamical strategy is proposed to modulate magnetic domain configurations, which is visualized by micromagnetic simulation and off-axis electron holography.
- The built-in electric field at the Fe-injected Ni/N-doped carbon aerogel (NF/NCA) interface, together with magnetic coupling, significantly enhances low-frequency electromagnetic wave absorption properties, as validated by an equivalent circuit model.
- NF@NCA composites offer additional radar stealth and thermal insulation performances, suitable for extreme temperature conditions; and a "robust" gradient metamaterial achieves 2–40 GHz absorption.

**Supplementary Information:**

The online version contains supplementary material available at 10.1007/s40820-025-01948-1.

## Graphical abstract

**Figure:**
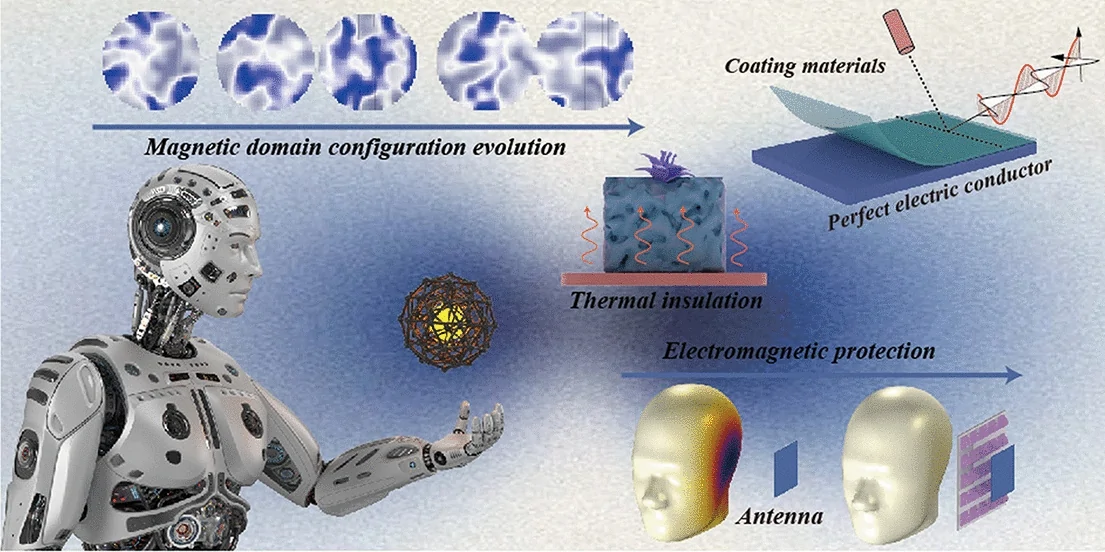

## Introduction

The rapid development of Bluetooth technology and 5G communication has substantially accelerated the adoption of artificial intelligence and wearable devices. However, this progress has also exacerbated electromagnetic (EM) interference issues, particularly in the ISM band (2.4–2.48 GHz) for Bluetooth devices, as well as the n77 (3.3–4.2 GHz), n78 (3.3–3.8 GHz), and n79 (4.4–5.0 GHz) bands for 5G communications. Such interference contributes to problems such as biological discomfort, EM signal disruptions, and information leakage [1–4]. Consequently, addressing EM interference and radiation pollution in the S-band (2–4 GHz) and C-band (4–8 GHz) is critical.

Magnetic materials with high permeability offer a promising solution because of their exceptional magnetic loss capabilities, enabling effective attenuation of low-frequency EM energy. Among these materials, magnetic nanoparticles (MNPs) exhibit superior magnetic coupling and stronger magnetic responses than bulk materials. However, the agglomeration and irregular distribution of MNPs remain significant barriers to achieving efficient magnetic performance [5, 6]. Importantly, the magnetic properties of materials are intrinsically linked to their magnetic structure, which can manifest as single-domain, vortex-state, or multi-domain arrangements. In particular, vortex domains, characterized by tilted spin states, form weak dipole–dipole interactions that stabilize the microstructure and prevent MNP aggregation [7]. Therefore, stabilizing the coexistence of multiple vortex domains is crucial for modulating magnetic domain configurations and achieving superior magnetic response characteristics.

A distinctive magnetic structure with multiple vortex domains can be achieved by controlling the composition, size, and spacing of MNPs based on the principle of energy minimization. This strategy enhances magnetic anisotropy and tunes the natural resonance frequency [8]. While previous studies have focused on developing magnetic compositions with high saturation magnetization, achieving sufficient permeability in the low-frequency range remains challenging owing to the Snoek limit [9, 10]. Enhancing magnetic coupling strength by adjusting MNP spacing offers a viable approach to overcoming this limitation. As MNPs move closer together, their external magnetic field lines interact, disrupting the original closed-loop structure and altering magnetic properties. These changes influence the intrinsic magnetic moments and the arrangement of magnetic domains, ultimately affecting macroscopic magnetic behavior [11]. Thus, precisely regulating MNP spacing presents an ideal pathway for tailoring magnetic properties systematically.

Various methods, such as chemical vapor deposition [12, 13], magnetic field induction [14, 15], electrospinning [16, 17], and Ostwald maturation [18, 19], have been explored for preparing highly dispersed magnetic nanostructures. However, these techniques often fail to achieve precise control over MNP spacing. To address this, a hydrogel precursor with a large network and periodic structure as well as multiple metal coordination sites is employed to selectively capture magnetic metal ions within a coordination thermodynamical system. After thermal reduction, the resulting materials exhibit controllable magnetic domain configurations, breaking the Snoek limit and achieving ideal saturation magnetization along with enhancing low-frequency electromagnetic wave absorption (EMWA).

Despite notable advances in magnetic structure design, the metallic behavior of MNPs often results in a skin effect, which limits low-frequency EMWA performance. By assembling magnetic units with other building blocks, spatial magnetic coupling can be achieved, transcending size limitation and improving intrinsic conductivity for superior wave impedance [20, 21]. The diverse electronic energy band structures of the two materials generate a surface work function differential, spontaneously establishing a built-in electric field. This enhances interfacial electron transport and facilitates charge migration under alternating EM fields, thereby significantly improving EM characteristics [22–24].

In this paper, we present a breakthrough method for controlling MNP spacing, enabling precise tuning of magnetic domain configurations through a novel thermodynamically controlled coordination strategy. This approach enhances magnetic coupling interactions, advancing EM attenuation and protection applications. Furthermore, a Fe-injected Ni (NF) alloy/N-doped carbon (NC) heterogeneous interface is fabricated via thermal reduction, increasing the interfacial work function difference and inducing electron migration polarization. Micromagnetic simulations and off-axis electron holography visualize the evolution mechanism of magnetic domain configurations, revealing the formation of a novel coupled vortex-domain state. This state effectively surpasses the Snoek limitation of ferromagnetic materials, significantly enhancing low-frequency permeability. Overall, this study advances our understanding of the relationship between magnetic domain configurations and EM characteristics; moreover, it offers a theoretical framework for designing high-performance low-frequency EMWA materials.

## Experimental Section

### Synthesis of N-doped Carbon Aerogel (NCA) Precursor

First, 46.8 mg of 1,3,5-tris(4-aminophenyl) benzene (TAPB), 26.8 mg of terephthaldicarboxaldehyde (PDA), and 10 mg of scandium trifluoromethanesulfonate (Sc(OTf)_3_) salt were added into a glass bottle containing 4 mL of N,N-dimethylformamide (DMF). The above mixed solution was further sonicated for 10 min using a 120 W ultrasonicator. After standing for several minutes, a gel formed. The gel was left to react for 24 h, after which the solvent was sequentially replaced with DMF, tetrahydrofuran (THF), acetone, ethanol, methanol, and water, forming a hydrogel.

### Synthesis of NF@NCA

A salt solution containing nickel and iron salts (Fe/Ni molar ratio = 1:10) was prepared in deionized water. This solution was injected into the hydrogel from top to bottom, allowing metal ions to adsorb onto the porous structure of the NCA hydrogel. The resulting mixture was freeze-dried overnight at a freezing temperature and vacuum degree of − 66 °C and 30 Pa, respectively, and subsequently pyrolyzed at 800 °C for 2 h under N_2_ flow to obtain NF@NCA-X. Metal salt concentrations of 0, 5, 10, 15, and 20% were used, and the corresponding materials were designated as NCA, NF@NCA-1, NF@NCA-2, NF@NCA-3, and NF@NCA-4, respectively.

EM simulations were conducted using the radio frequency domain module of COMSOL Multiphysics software, employing the finite element method. The fluctuation equation of the EM field is [25, 26]:

1 $$\nabla \times \mu_{{\mathrm{r}}}^{ - 1} \left( {\nabla \times {\boldsymbol{E}}} \right) - k_{0}^{2} \left( {\varepsilon_{{\mathrm{r}}} - \frac{j\sigma }{{\omega \varepsilon_{0} }}} \right){\boldsymbol{E}} = 0$$

here *ε*_*r*_ and *μ*_*r*_ are the relative permittivity and permeability, ***E*** is the electric field vector, and *k*_*0*_, *σ*, *ω*, and *ε*_*0*_ represent the wave vector, conductivity, angular frequency, and free-space permittivity, respectively. The electric field loss and power loss density of NF@NCA were examined using space EM field simulations.

When EMWs propagate through biological tissue (BT), the BT absorbs the EMW energy. The interaction of EMWs with BT is quantified by the *SAR*, which is described by the following expression [27]:

2 $$SAR = \frac{\sigma }{\rho }\left| {\boldsymbol{E}} \right|^{2}$$

here *σ* and *ρ* represent the electrical conductivity and density of BT, respectively.

However, the average *SAR* is a more practical quantity that can be defined as the ratio of the power absorbed by BT to its weight. This value can be obtained by integrating the following equation:

3 $$SAR_{{{\mathrm{avg}}}} = \frac{1}{V}\int_{V} SAR dV = \frac{1}{V} \int_{V} \frac{\sigma }{\rho }\left| {\boldsymbol{E}} \right|^{2} dV$$

here *V* is the BT volume. The mass-averaged *SAR* is usually calculated for a 1 g sample (*SAR*_*1g*_).

Micromagnetic simulations were performed using the open-source software Mumax3, which is based on GPU acceleration. Dynamic physical processes were modeled using the Landau–Lifshitz–Gilbert equation [28]:

4 $$\frac{{d{\boldsymbol{m}}}}{dt} = - \gamma {\boldsymbol{m}} \times {\boldsymbol{H}}_{{{\mathrm{eff}}}} + \alpha {\boldsymbol{m}} \times \frac{{d{\boldsymbol{m}}}}{dt}$$

here ***m*** is the unit vector of local magnetization, *γ* is the gyromagnetic ratio, ***H***_***eff***_ is the effective magnetic field, and *α* is the Gilbert damping parameter.

### Characterization

The molecular structure, crystalline structure, porosity, surface chemical composition, magnetic properties, microstructure, and morphology of the prepared samples are characterized by Fourier transform infrared (FT-IR) spectroscopy (Bio-Rad FTS-40, USA), X-ray diffraction (XRD; Bruker D8 Advance A25), Brunauer–Emmett–Teller (BET) analysis (BELSORP-MAX), X-ray photoelectron spectroscopy (XPS; Thermo Fisher ESCALAB Xi +), vibrating sample magnetometry (VSM; Quantum Design DynaCool), transmission electron microscopy (TEM; Talos F200X), high-resolution TEM (HRTEM), energy dispersive spectroscopy (EDS), and field emission scanning electron microscopy (FE-SEM; FEI Nova NanoSEM450). Thermal conductivity is measured with HotDisk thermal conductivity meter, which is based on the transient plane source method. Additionally, a thermal imaging camera (E85, FLIR) is utilized to evaluate the thermal insulation performance of NF@NCA-3.

## Results and Discussion

### Fabrication and Characterization

**Fig. 1:**
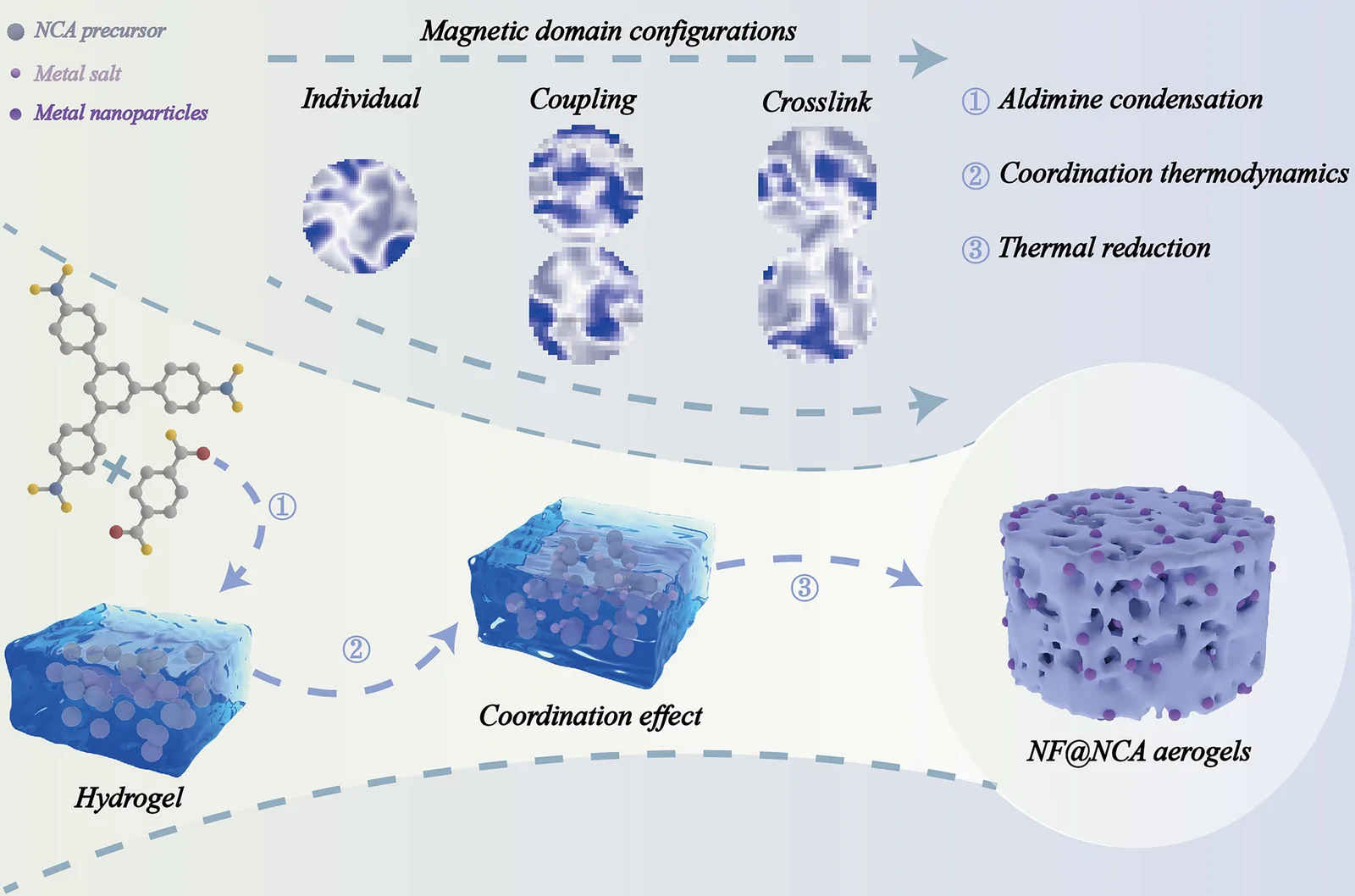
Magnetic response modulation strategy for coordination thermodynamically controlled NF@NCA composites

The precise tuning of MNP spacing is achieved through a novel thermodynamically controlled coordination strategy. According to thermodynamic theory, the coordination reaction, accompanied by an enthalpy-decreasing process, renders free metal cations unstable, which drives the formation of coordination [29]. As ligands approach central transition metal ions, the static electric field disrupts the *d*-orbitals of the metal ions, causing orbital splitting. Electrons preferentially occupy low-energy *d*-orbitals, reducing the energy of the overall system. Additionally, based on the rule of “soft and hard acids and bases,” the *d*-orbitals of Ni and Fe atoms readily hybridize with the *p*-orbitals of N atoms, forming stable coordination bonds [30]. Specifically, TAPB, with three amino groups, is selected to react with PDA via a simple aldimine condensation, constructing a hydrogel network with fixed and periodic metal coordination sites. Subsequent metal salt impregnation introduces Ni and Fe ions uniformly into the hydrogel through bottom-up coordination strategy, further stabilizing the network. This directional coordination mechanism ensures that metal ions are introduced in a predetermined uniform dispersion manner, laying the foundation for precise structure control. The final products of NF@N-doped carbon aerogels (NCA) are obtained by further freeze-drying and followed by high-temperature thermal reduction (Fig. S1). By modulating the concentration of the magnetic metal salt solution, the magnetic domain configuration is precisely controlled (Fig. 1).

**Fig. 2:**
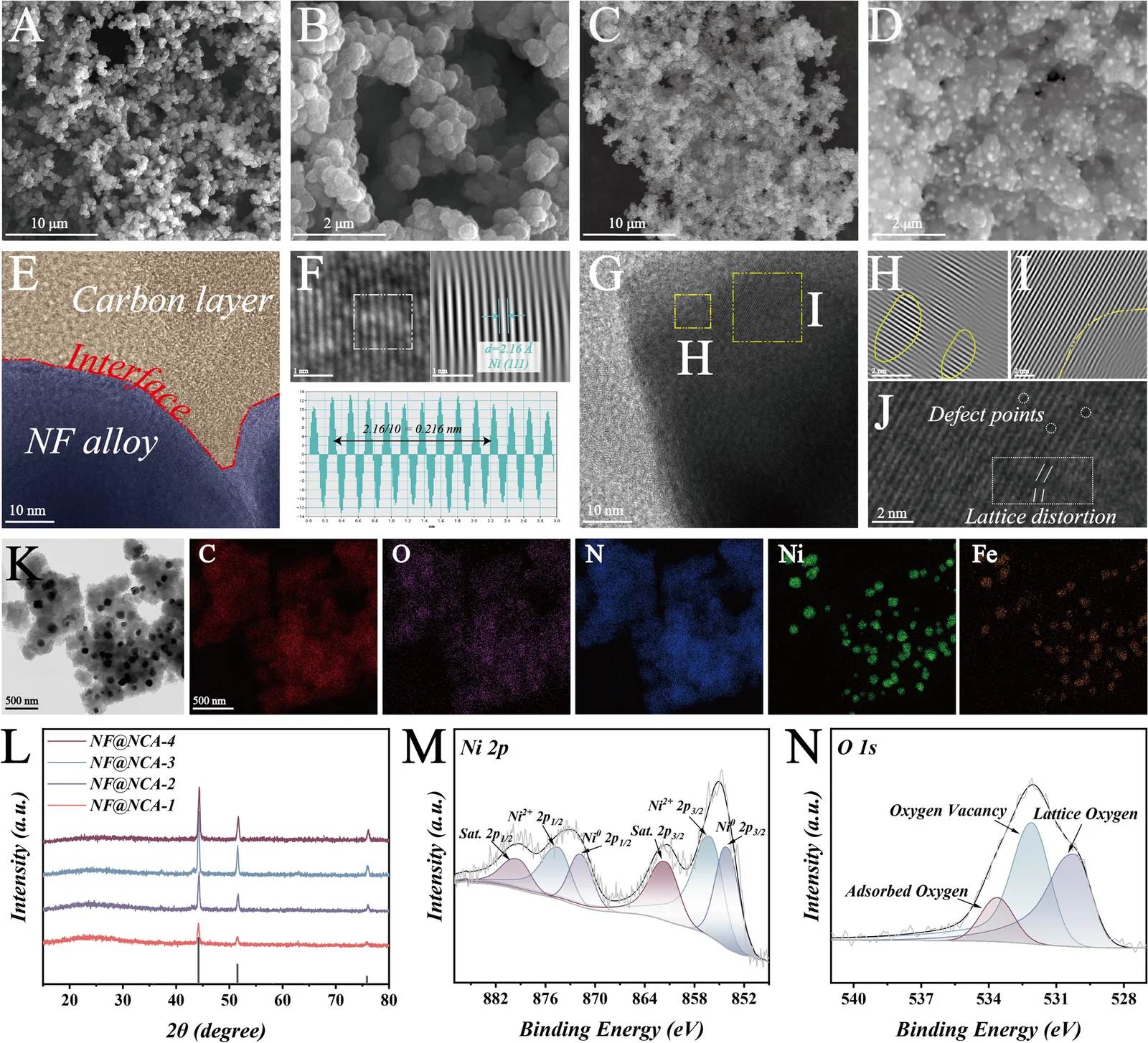
Morphological and physicochemical characterization of NF@NCA composites. SEM images of **a–b** NCA and **c–d** NF@NCA-3. **e–j** HRTEM images of NF@NCA-3. **k** TEM and EDS images of NF@NCA-3. **l** XRD pattern of NF@NCA composites. **m** Ni 2*p* XPS spectra, **n** O 1*s* XPS spectra of NF@NCA-3

The morphology of the synthesized NF@NCA samples was characterized using scanning electron microscopy (SEM) and transmission electron microscopy (TEM). Figure 2a and b shows that the NCA spheres exhibit uniform size, while the NF nanoparticles (Fig. 2c and d) grow on the NCA spheres via a spontaneous diffusion strategy. High-resolution TEM (HRTEM) images and lattice spacing measurements (Fig. 2e and f) reveal an average lattice spacing of 0.216 nm, corresponding to the (111) plane of Ni crystals. The incorporation of Fe induces lattice distortion due to its larger atomic radius compared to Ni, resulting in an increased lattice spacing [31]. The HRTEM image (Fig. 2e) also demonstrates the formation of a magnetic-carbon heterogeneous interface between NF nanoparticles and graphitic carbon, which enhances polarization loss. Additionally, Fig. 2g–j shows the presence of several lattice defects in the NF@NCA composites, including point defects and lattice distortions. These defects interact with EMWs to induce dipole polarization, effectively dissipating EM energy [32, 33]. Energy-dispersive X-ray spectroscopy (EDS) mapping (Fig. 2k) confirms the uniform distribution of NF nanoparticles on the N,O co-doped carbon substrate.

Through the thermodynamically controlled coordination strategy, independent control over MNP size is achieved, with sizes ranging from 45.62 to 279.73 nm (Fig. S4d–g). Importantly, the embedding of NF alloys in the NCA carriers prevents agglomeration, even with metal salt loadings up to 20 wt%. As metal salt content increases, the average MNP size grows, while the spacing between MNPs decreases, enabling precise regulation of MNP spacing and magnetic response behavior. Figure S6a illustrates the N_2_ adsorption–desorption isotherms of the NF@NCA composites, where distinct type-IV hysteresis curves indicate a decrease in specific surface area and pore volume with increase in metal salt concentration (Fig. S6b). The incorporation of NF nanoparticles modulates the magnetic domain configuration, enhancing macroscopic magnetic coupling and resonance capabilities. The resulting magnetic network interferes effectively with EMWs. X-ray diffraction (XRD) patterns of NF@NCA composites (Fig. 2l) display characteristic peaks at 44.3°, 51.6°, and 76°, corresponding to the (111), (200), and (220) planes of Ni (PDF#03–65-0380). The broad peak at 23.5° is attributed to the (002) plane of graphitic carbon, confirming the successful synthesis of NF@NCA composites. High-resolution Ni 2*p* XPS spectra (Fig. 2m) reveal the presence of Ni^0^, indicating the formation of Ni [34]. The O 1*s* spectrum (Fig. 2n) shows a prominent peak at 532.1 eV, indicative of oxygen vacancies, confirming the presence of abundant carriers within the material, which contribute to enhanced conductivity [35, 36].

### EMWA Performance

**Fig. 3:**
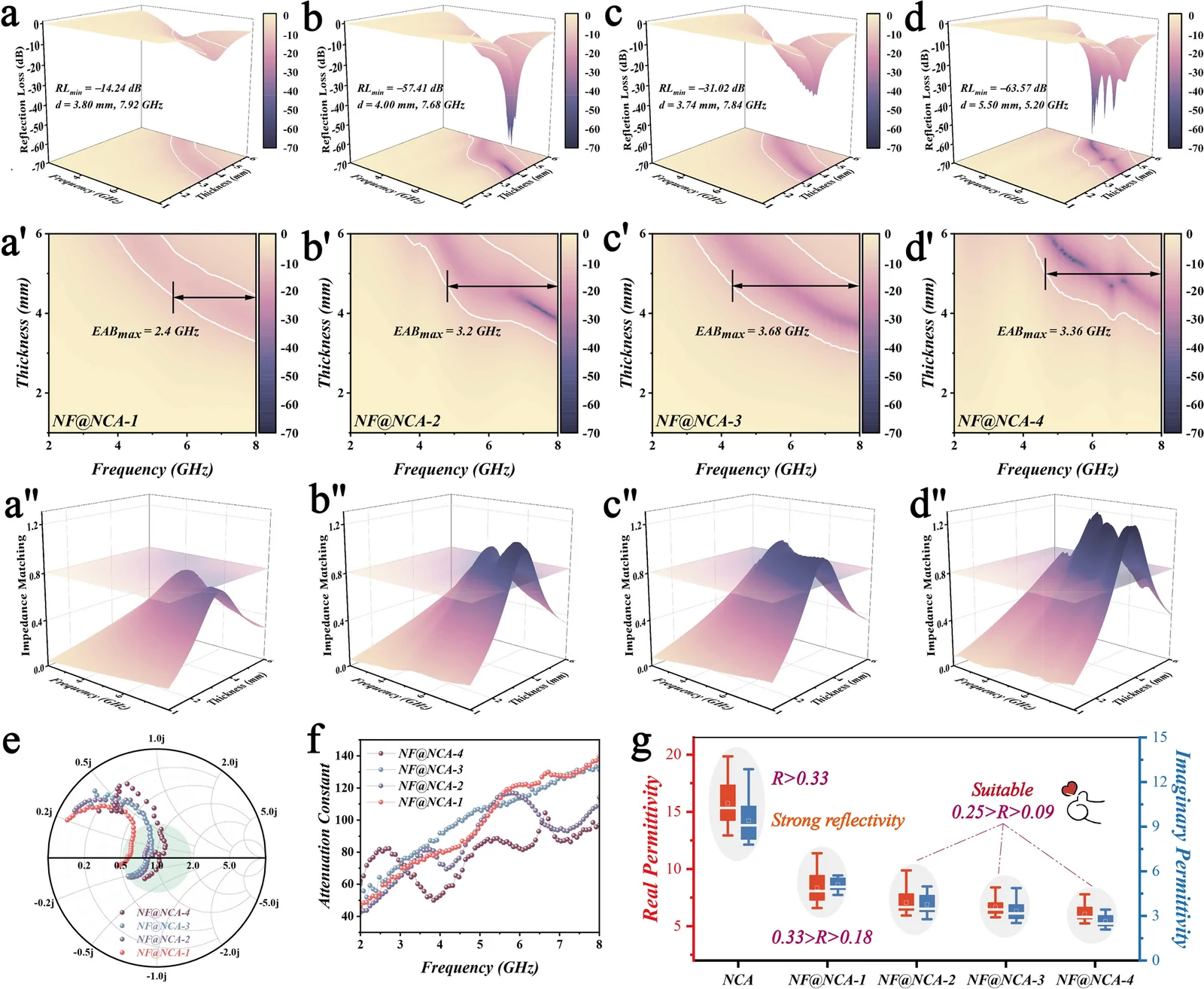
EMWA performance of NF@NCA composites. 3D *RL* mappings, 2D EAB mappings, and 3D impedance matching diagrams for **a–a"** NF@NCA-1, **b–b"** NF@NCA-2, **c–c"** NF@NCA-3, and **d–d"** NF@NCA-4. **e** Smith charts at a thickness of 4.69 mm. **f** Attenuation constants of NF@NCA composites. **g** Complex permittivity of NCA and NF@NCA composites

The EMWA performance of NF@NCA composites was evaluated based on reflection loss (*RL*) and effective absorption bandwidth (EAB) in the 2–8 GHz range. The 3D *RL* mappings indicate that NF@NCA-1 exhibits the lowest EMWA performance, with an *RL*_*min*_ value of − 14.24 dB at a thickness of 3.80 mm (Figs. 3a and S8a). In contrast, NF@NCA-2 achieves an *RL*_*min*_ of − 57.41 dB at 4.00 mm thickness (Figs. 3b and S8b), while NF@NCA-3 shows effective absorption of − 31.02 dB with a reduced thickness of 3.74 mm (Figs. 3c and S8c). Notably, NF@NCA-4 achieves the highest *RL*_*min*_ of − 63.57 dB at 5.50 mm thickness (Figs. 3d and S8d). The effect of MNP spacing (Fig. 3a'–d') on EAB is evident, with moderate spacing improving magnetic loss ability and impedance matching. NF@NCA-3 demonstrates a maximum EAB of 3.68 GHz, covering nearly the entire C-band (Fig. 3c'), making it highly suitable for low-frequency EMWA applications, including the mitigation of 5G radiation pollution.

Impedance matching is a critical factor for EM energy dissipation. An impedance matching value close to 0.8 indicates efficient penetration of EMWs into the absorber. Figure 3a"–d" shows that NF@NCA-3 achieves optimal impedance matching at all tested thicknesses, surpassing the performance of other samples. In the Smith chart, the *Z*_*in*_ within the green circle indicates that the *RL* values are below −10 dB. More points within the green region suggest better impedance matching and a broader EAB. Figure 3e further confirms that NF@NCA-3 exhibits a higher proportion of points within the effective absorption region. Thus, the superior impedance matching characteristics of NF@NCA-3 result in a broader EAB. The attenuation constant, another key parameter for evaluating EM energy dissipation, is high in NF@NCA composites, signifying a strong ability to convert EM energy into other forms of energy (Fig. 3f). Reflection coefficients (*R*), calculated as described in Equation S8, further support the excellent EMWA performance. *R* values below 0.3 suggest effective EMW absorption, while higher *R* values (above 0.3) imply excessive conductivity, leading to a skin effect that hinders EMW absorption (Fig. 3g).

### Electromagnetic Attenuation Mechanism

**Fig. 4:**
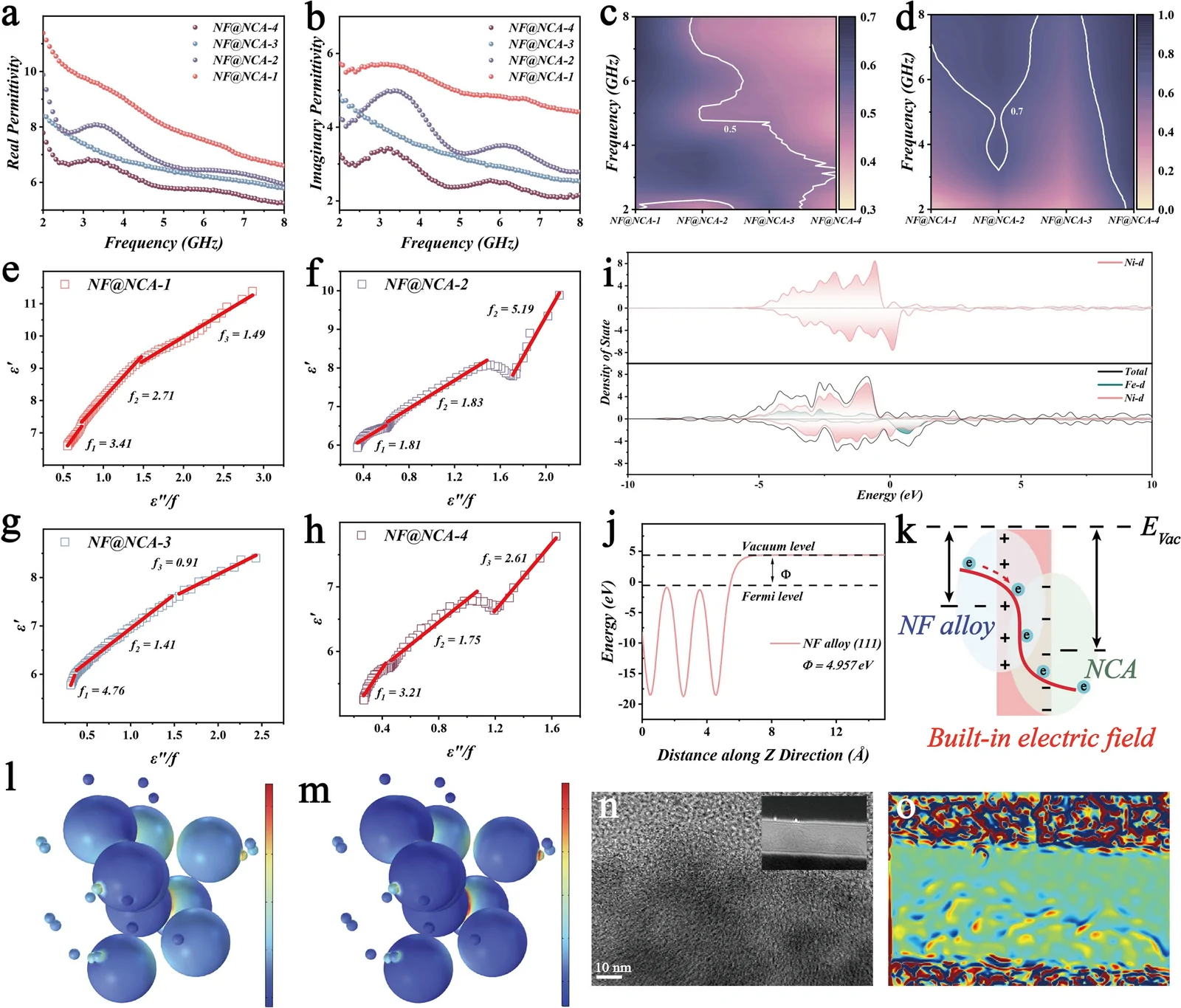
Dielectric loss mechanisms of NF@NCA composites. **a** Real part and **b** imaginary part of permittivity, **c** dielectric loss tangent, **d** polarization percentage, **e–h** polarization relaxation behavior of NF@NCA composites. **i** DOS for Ni and NF alloy. **j** Work function of the NF alloy (111) plane. **k** Schematic of the built-in electric field. **l** Electric field loss. **m** Power loss density. **n** Electron hologram with TEM inset and **o** charge density mapping

To better understand the EMWA performance of NF@NCA composites, it is crucial to analyze the loss mechanisms. The real (*ε'*) and imaginary (*ε"*) parts of permittivity for the NF@NCA composites are shown in Fig. 4a and b. Both parts decrease with increase in frequency, a trend most pronounced in NF@NCA-1. Specifically, the *ε'* and *ε"* values for NF@NCA-1 range from 11.38 to 6.59 and from 5.72 to 4.41, respectively. As the metal content increases, both parts of permittivity decline. For NF@NCA-2, NF@NCA-3, and NF@NCA-4, *ε'* values reduce from 9.88 to 5.93, 8.39 to 5.77, and 7.78 to 5.24, respectively, while *ε"* values decrease from 4.98 to 2.78, 4.86 to 2.52, and 3.42 to 2.09, respectively. This phenomenon is attributed to MNPs overriding dipole centers, which shifts the polarization [37]. NF@NCA-2 and NF@NCA-4 exhibit two prominent resonance peaks, indicating significant internal polarization loss conducive to the strong EMWA. The dielectric loss capability, quantified through the dielectric loss tangent (*tanδ*_*ε*_ = *ε"*/*ε'*), is shown in Fig. 4c. All samples demonstrate excellent dielectric loss capability. In addition, the contribution of *ε*_*p*_*"* is quantified, and the ratio *ε*_*p*_*"*/*ε"* is defined as the polarization percentage [38]. As shown in Fig. 4d, NF@NCA-2 and NF@NCA-4 exhibit superior polarization loss capabilities.

The enhancement of polarization loss is further analyzed using Equation S7 to extract relaxation times for different polarization behaviors. The linear relationship between *ε'* and *ε"*/*f* suggests that dielectric loss correlates with polarization relaxation. Under an external EM field, dipole orientation shifts occur. Multiple fitted lines with varying slopes in Fig. 4e–h confirm the existence of multi-polarization relaxation mechanisms in the NF@NCA composites [39, 40]. The similar physicochemical properties of Ni and Fe allow for adjustments to their electronic structures and dielectric properties. The high electronegativity of Ni facilitates efficient electron transfer from Fe to Ni, inducing dipole polarization [41]. As shown in Fig. 4i, density of states (DOS) analysis reveals that the Ni-3*d* band in NF alloy is populated by electrons from the Fe-3*d* band, significantly increasing the electron density compared to pure Ni. This electron-rich Ni-3*d* band enhances charge transfer under EM fields. Additionally, Fe incorporation shifts the center of the D-band from − 1.567 to − 1.629 eV, increasing electron density and forming space electric dipoles, further boosting polarization loss [42]. Density functional theory (DFT) calculations indicate that the work function (Φ) of the NF alloy (111) plane is 4.957 eV (Fig. 4j), while Φ for the NCA substrate is 5.765 eV [43]. When NF alloy comes into contact with NCA, because of the difference in work function, the electrons spontaneously flow from the NF alloy with low work function to the NCA substrate with high work function until the Fermi levels of both equilibrate (Fig. 4k). This charge transfer process forms a space charge region at the interface: the NF side, having lost the electrons, forms a hole accumulation layer with positive charges, causing the band structure to bend upward; the NCA side, having gained the electrons, forms an electron accumulation layer with negative charges, causing the band structure to bend downward. The electrostatic interaction between the positive and negative charge layers generates a built-in electric field directed from NF toward NCA. Charge transferring from NF nanoparticles to the NCA substrate leads to an intermediate Φ value owing to spontaneous alignment, resulting in a space charge region that enhances interface polarization [44].

Interface polarization at the heterogeneous interfaces is visualized using COMSOL Multiphysics 6.2. The enhanced polarization primarily occurs at the NF–NF, NCA–NCA, and NCA–NF interfaces (Fig. 4l). The power loss density (Fig. 4m) aligns with the electric field loss region, further confirmed by electron holography. TEM and charge density maps (Fig. 4n, o) reveal a high concentration of positive and negative carriers around heterointerfaces, which contributes to interface polarization and improves EM dissipation performance.

**Fig. 5:**
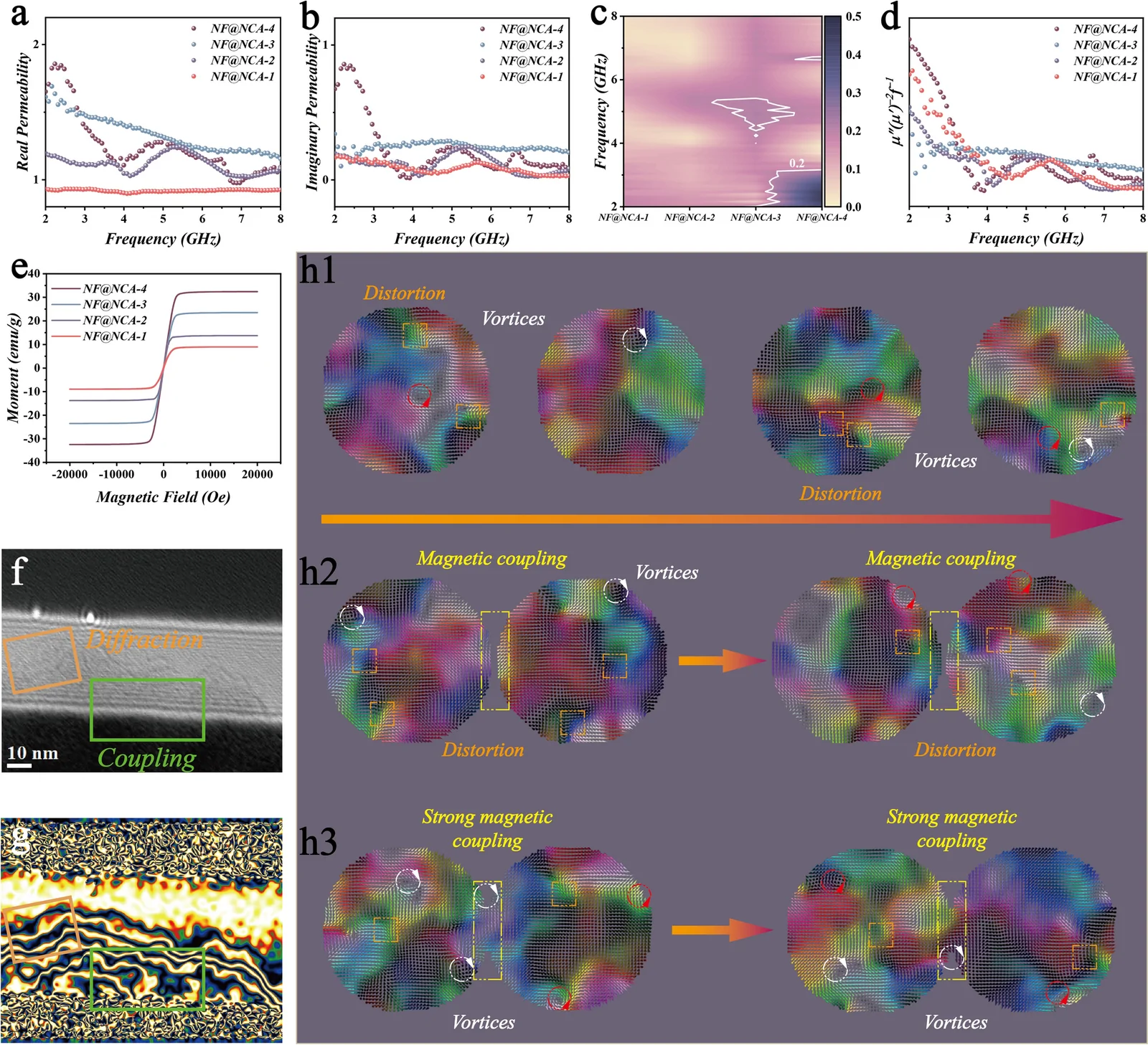
Magnetic loss mechanism. **a** Real part and **b** imaginary part of complex permeability. **c** Magnetic loss tangent. **d** Eddy current loss value. **e** Room-temperature magnetic hysteresis loops of NF@NCA composites. **f** Off-axis electron holograms of sliced NF@NCA-3 and **g** corresponding magnetic flux lines. Micromagnetic simulations of **h1** individual, **h2** coupled, and **h3** crosslinked magnetic configurations

The tailored magnetic domain configuration of NF@NCA composites enables tunable magnetic response characteristics and enhanced magnetic dissipation capabilities. The real (*µ'*) and imaginary (*µ"*) parts of permeability represent magnetic energy storage and dissipation loss, respectively. As shown in Fig. 5a, b, *µ'* increases with higher MNP content, and the magnetic performance of NF strongly depends on frequency. Specifically, the *µ'* values for NF@NCA-1, NF@NCA-2, NF@NCA-3, and NF@NCA-4 range from 0.93 to 0.89, 1.24 to 1.02, 1.69 to 1.15, and 1.85 to 0.97, respectively. Correspondingly, the *µ"* values vary with frequency from 0.17 to 0.02, 0.24 to 0.02, 0.34 to 0.10, and 0.85 to 0.01, respectively. Notably, the magnetic domain design enhances magnetic loss capability. NF@NCA-3 with optimized MNP spacing breaks the Snoek limitation successfully and demonstrates higher permeability than NF@NCA-4 over a large frequency range. Figure 5c shows that NF@NCA-4 has the highest *tanδ*_*µ*_ value, indicating the strongest magnetic loss capability, consistent with *µ"* trends*.* Eddy current effects, though present, are overshadowed by multiple resonance peaks (Fig. 5d), which suggest that natural resonance is the dominant magnetic loss mechanism in the NF@NCA system. The imaginary permeability (*µ"*) is closely linked to the intrinsic saturation magnetization (*M*_*s*_) of magnetic absorbers. Using the S–W approximation, *M*_*s*_ values for NF@NCA-1, NF@NCA-2, NF@NCA-3, and NF@NCA-4 are calculated as 8.96, 13.75, 23.52, and 32.41 emu g^−1^, respectively (Fig. S11a). The degree of magnetic exchange coupling is determined from the switching field distribution (SFD) curve, calculated as the first derivative of the demagnetization curve (Fig. S11b) [45]. A single peak at zero external field indicates synchronous magnetic coupling, suggesting well-coupled adjacent metallic MNPs. Moreover, the saturation magnetization is enhanced, forming an extensive magnetic coupling network. NF@NCA-4 demonstrates the strongest exchange coupling ability. MNP spacing optimization enhances *M*_*s*_ by improving magnetic exchange coupling, ensuring high permeability and superior magnetic energy storage and dissipation in low-frequency regions.

Magnetization behavior, exchange forces, and magnetic resonance of MNPs are strongly influenced by nanoscale particle size. Individual MNPs not only radiate flux lines for macroscopic magnetic coupling but also extend flux lines into free space for long-range magnetic diffraction [46]. The magnetic response behavior of NF@NCA-3 is visualized through off-axis electron holography. As shown in Fig. 5f and g, internal magnetic moments generate periodic spin precession under an external EM field, producing mutual coupling effects. Closed magnetic flux lines confirm a subtle magnetic coupling network among ferromagnetic NF particles, while magnetic flux lines extending into free space generate long-range diffraction resonance. This unique structure expands the magnetic response region, creating a magnified magnetic dissipation system.

The dynamic magnetic moment movement in NF@NCA was investigated using micromagnetic simulations to analyze the magnetic moment dynamics and coupling behaviors of various magnetic domain configurations under an alternating magnetic field. This approach further explored the dynamic evolution process of magnetic domains [47, 48]. Under an external magnetic field of 5.2 GHz, the magnetic response behavior of individual MNPs evolves significantly (Fig. 5h1). The vortex nuclei within the vortex-domain vibrate around their geometrical centers and transform into transverse domain walls at the boundaries, which subsequently reform vortex nuclei with opposite polarity. The evolution of the magnetic moment lags behind that of the external magnetic field vector, leading to an irreversible magnetic hysteresis phenomenon. The formation, migration, distortion, and disappearance of magnetic vortex domains exhibit significant domain wall migration, facilitating magnetic energy dissipation. Additionally, simulations of MNPs with varying spacing are performed to explore the response of diverse magnetic domain configurations. Magnetic coupling is observed when adjacent MNPs have aligned or opposing magnetic moments at their boundaries. A significant coupling effect is evident in Fig. 5h2. Compared to other models, the configuration shown in Fig. 5h3 demonstrates more intense magnetic moment oscillations under an alternating magnetic field. Magnetic domains couple at the junctions, forming new magnetic vortex states with pronounced magnetic coupling response behavior. Consequently, NF@NCA-3 and NF@NCA-4, which feature moderate MNP spacing, form dense magnetic coupling networks that effectively interact with incident EMWs. The enhanced intrinsic magnetic properties and increased magnetic coupling synergistically improve magnetic loss capabilities. Notably, NF@NCA-3, with superior impedance matching, demonstrates enhanced EMWA performance.

To systematically elucidate the EM loss mechanism, a detailed prototype model was developed (Fig. S12). The model consists of a conductive module (upper) and a thermal conversion module (lower). The conductive module collects and converts incident EM energy into electric field energy, which is subsequently dissipated as electrical and thermal energy via the respective modules. Dielectric loss is represented by a parallel circuit of ideal capacitance (*C*) and effective resistance (*R*_*e*_), while magnetic loss is modeled as a series circuit of ideal inductance (*L*) and effective resistance (*R*_*m*_). These two modules are combined into a series circuit based on physical principles [49, 50]. Figure S13a–c illustrates the contributions of polarization relaxation, conduction, and magnetic loss to EM dissipation. As MNP spacing decreases, the number of space charges increases, strengthening the built-in electric field strength and enhancing dielectric loss capabilities. The built-in electric field also facilitates electron transfer from NF to the NCA substrate, suppressing metallic conduction behavior and mitigating skin effects. Natural resonance and MNP coupling enable significant magnetic energy conversion in the low-frequency region. Despite the absence of a significant decrease in magnetic loss across the tested frequencies, breaking the Snoek limit indicates effective magnetic energy dissipation. Differences in magnetic loss contributions among the samples are attributed to the influence of magnetic domain configurations on magnetic properties. The values of magnetic loss are a little lower than those of the dielectric loss, and the overall loss ability aligns with the trend in magnetic loss behavior, which demonstrates the important contribution of magnetic loss in the low-frequency region (Fig. S13d).

**Fig. 6:**
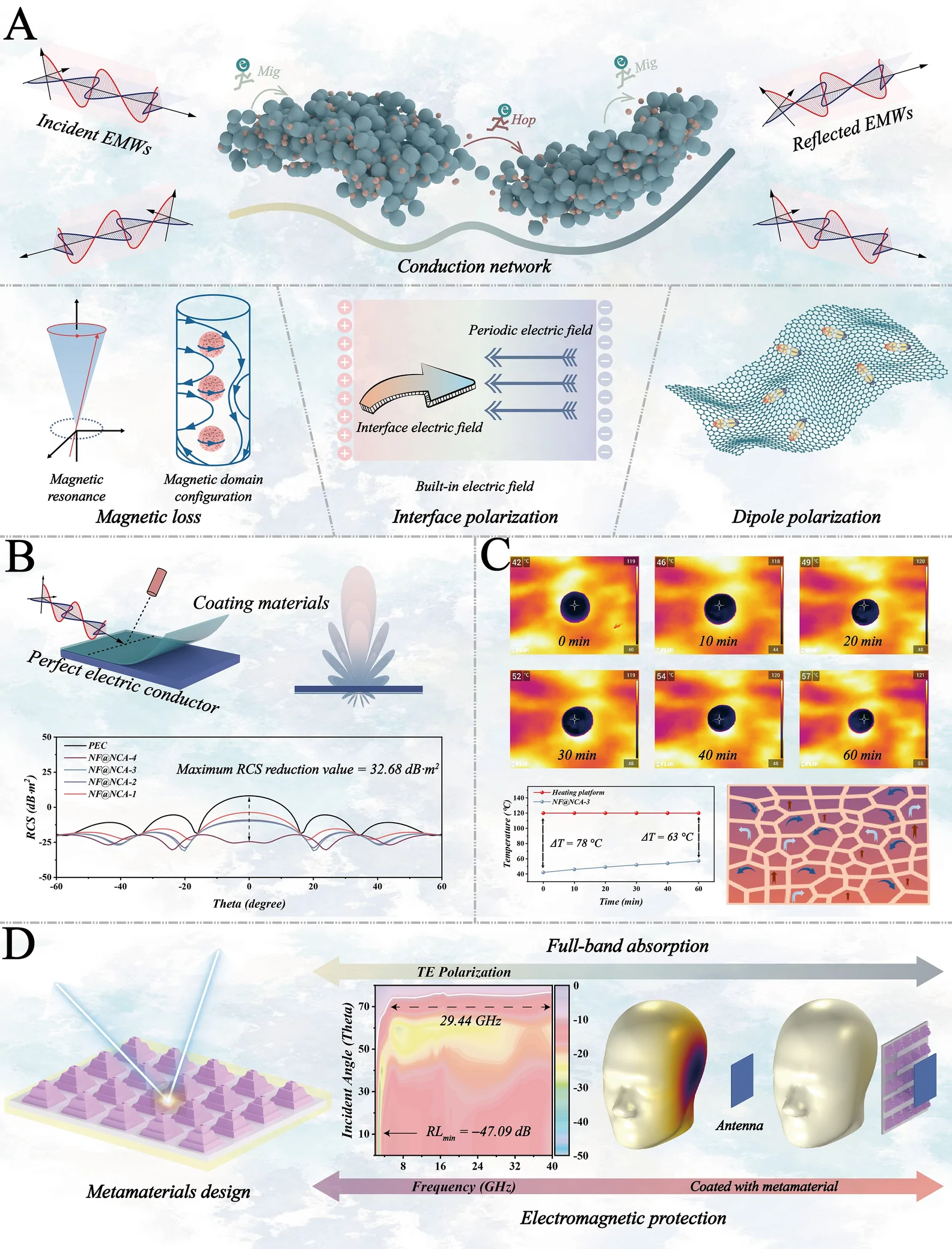
EM loss mechanism and multifunctional performance exploration. **a** EMWA mechanism of NF@NCA composites. **b** Radar stealth properties of NF@NCA composites. **c** Thermal insulation performance of NF@NCA-3. **d** Gradient metamaterial design showcasing EMW attenuation properties for Bluetooth devices

Based on the above analysis, the enhanced EMWA performance of NF@NCA composites is closely linked to the built-in electric field and magnetic domain configurations (Fig. 6a). The efficient conduction network, established by the intrinsic conductivity of the materials, facilitates electron migrating and hopping, converting EM energy into thermal energy and resulting in significant conduction loss. Furthermore, numerous electric dipoles induced by oxygen vacancies, N-doping, and defects rotate and vibrate in the applied EM field, driving polarization relaxation and further attenuating EM energy. A spontaneous built-in electric field formed at the NF/NCA interface modifies electron transfer, electron density, and the energy band structure near the Fermi level, enhancing space charge relaxation. The built-in electric field vector aligns with or opposes the applied electric field vector, eventually achieving a new dynamic equilibrium under the driving force. This results in effective interface polarization, significantly boosting EM energy attenuation. Similarly, the magnetic domain configurations demonstrate varied magnetic responses in the external EM fields due to differences in MNP spacing. A newly observed magnetic vortex state at the boundary strengthens magnetic energy attenuation. Additionally, the increased permeability in the low-frequency region greatly enhances magnetic loss and amplifies the strength and range of effective fields generated by the magnetic domain configuration.

### Multifunctional Performance

Radar cross section (RCS) simulations were conducted to evaluate the EM attenuation capability of the NF@NCA composites under practical far-field conditions [51]. A two-layer square plate model is constructed for the calculations, with the thicknesses of the perfect electric conductor (PEC, 200 × 200 mm^2^) and coating materials (200 × 200 mm^2^) set to 2 and 5.5 mm, respectively (Fig. 6b). The simulation frequency is set at 5.2 GHz. The pure PEC exhibits the strongest EMW scattering values, which is detrimental to radar stealth. Therefore, the far-field responses of the composites are evaluated by comparing their RCS values to that of the pure PEC reference. At an incident angle of 0°, the scattering values of all the NF@NCA samples are significantly weaker than those of the pure PEC, indicating superior radar wave absorption (Fig. S14a–d). Notably, NF@NCA-4 achieved the maximum RCS reduction value of 32.68 dB m^2^, consistent with its superior EMWA performance. These findings provide a solid foundation for the practical application of NF@NCA composites in radar stealth technologies.

Effective thermal insulation materials hold immense potential for applications in harsh environments [52]. To assess the thermal insulation performance of NF@NCA-3, the aerogel is placed on a hot plate maintained at 120 °C (Fig. 6c). After 10 min, the temperature of the aerogel increased modestly from 42 to 46 °C, reaching 54 °C after 40 min and stabilizing at 57 °C after 60 min. Throughout this process, the aerogel structure remained intact, demonstrating high thermal stability. The absolute temperature difference ΔT between the aerogel and the heat source exceeds 63 °C. The thermal insulation mechanism of NF@NCA-3 composites involves heat conduction, convection, and radiation. The porous structure of the aerogel significantly enhances its ability to trap air, while the thermal conductivity of the composite (0.045 W m^−1^ K^−1^) closely approximates that of air (0.026 W m^−1^ K^−1^), effectively reducing heat conduction. Additionally, the carbon network extends the heat conduction path, further decreasing thermal conductivity, while the curved pathways of the aerogel minimize thermal convection. The high porosity also reduces thermal radiation from the solid phase, collectively improving thermal insulation performance. These findings demonstrate that NF@NCA-3 composites exhibit excellent thermal insulation capabilities, which are suited for the application under extreme conditions, such as ultra-high and ultra-low temperatures.

The Planck–Rozanov limit hinders broadband absorption in single-layer EMWA materials, posing challenges for their application [53]. To address this, the most representative composite of NF@NCA-3 is selected for structural optimization. Notably, an innovative gradient structure design can generate multiple EM responses, while adjusting the thickness of each layer in the multilayer structure can enable interferometric phase cancelation. This process creates multiple EMWA peaks that overlap, achieving ultrabroadband EMWA. Herein, a simple gradient metamaterial with a honeycomb-perforated design is devised (Fig. S16l). Consequently, ultrabroadband absorption is achieved, covering the 2 to 40 GHz frequency range, with an *RL*_*min*_ of − 47.09 dB at 2.76 GHz. A detailed analysis of the electromagnetic properties of the metamaterial reveals that the electric field is concentrated at interlayer edges, while the magnetic field localizes at the bottom layer (Fig. S17a, b). The phase difference of π/2 between these fields indicates the formation of standing waves within the material. Power loss density analysis further confirms that the strongest absorption peak at 2.76 GHz arises from the quarter-wavelength diffraction resonance of the bottom layer and the honeycomb structure (Fig. S17c).

In complex EM environments, robustness against varying angles and polarization modes of incident EMWs is crucial. Simulations demonstrate that the metamaterial absorber maintains stable performance in transverse electric (TE) polarization, with an EAB exceeding 37 GHz at incident angles below 60°. Even at 70°, the EAB remains close to 30 GHz (Figs. 6d and S17d). Similarly, in transverse magnetic (TM) polarization, the metamaterial exhibits consistent absorption for angles up to 50°, with an EAB exceeding 28 GHz in all cases (Fig. S17e, f). The frustum structure reduces the effective incident angle between EMWs and the surface of the metamaterial, while the honeycomb-perforated top layer increases multiple EMW reflections and scatterings.

This robust metamaterial with ultrabroadband EMWA capabilities is also suitable for EMWA scene. Given the proliferation of 5G and smart devices, the increasingly complex EM environment poses risks to human health. The average specific absorption rate (*SAR*_*avg*_), a measure of EM radiation energy absorbed by a unit mass of material per unit time, is simulated by a 2.45 GHz antenna model representing Bluetooth devices in COMSOL software (Fig. S18) [54]. The model without protection (left, Fig. 6d) shows severe EM radiation near the antenna, indicated by the dark region. By contrast, the model with metamaterial protection (right) exhibits negligible radiation, demonstrating its effectiveness in attenuating EM energy and highlighting its potential for application in EMWA.

## Conclusions

In summary, NF@NCA composites with controllable magnetic domain configurations were successfully synthesized using an ingenious and periodic coordination thermodynamically strategy. By fine-tuning the metal ion concentration, the spacing between MNPs was precisely regulated, enabling modulation of magnetic domain configurations. This resulted in superior magnetic coupling behavior, effectively breaking through the Snoek limitation and significantly enhancing the permeability and EMWA performance in the low-frequency range. Furthermore, the spontaneous built-in electric field formed at the NF/NCA interface improved space charge relaxation in the external EM field due to the disparity in work functions, thereby substantially boosting polarization loss characteristics. The far-field EM response confirmed the enhanced EM attenuation performance of the composites under practical conditions, while their thermal insulation properties demonstrated stable functionality in harsh environments. The robust gradient metamaterial design achieved ultrabroadband EMWA performance spanning 38 GHz (2–40 GHz), fully covering the Bluetooth and 5G frequency bands. EM simulations further validated the excellent EM protection properties of the metamaterial. This study demonstrated the precise modulation of magnetic properties through a macroscopic magnetic space, providing valuable insights into the development of low-frequency high-performance, ferromagnetic EMWA materials.

## Supplementary Information

Below is the link to the electronic supplementary material.

Supplementary file1 (DOCX 22589 KB)
