# Supplementary material for: Coordination Thermodynamic Control of Magnetic Domain Configuration Evolution toward Low-Frequency Electromagnetic Attenuation
Source: Nanomicro Lett. 2026 Jan 8;18:104. doi: 10.1007/s40820-025-01948-1 (PMC12779889; doi:10.1007/s40820-025-01948-1)
Supplement: Supplementary file 1 — Supplementary file1 (DOCX 22589 KB) [file 40820_2025_1948_MOESM1_ESM.docx]

Supporting Information for

**Coordination Thermodynamic Control of Magnetic Domain Configuration Evolution toward Low-Frequency Electromagnetic Attenuation**

Tong Huang^1,†^, Dan Wang^1,†^, Xue He^2,†^, Zhaobo Feng^1,†^, Zhiqiang Xong^1^, Yuqi Luo^1^, Yuhui Peng^3,^*, Guangsheng Luo^4,^*, Xueliang Nie^5,^*, Mingyue Yuan^6^, Chongbo Liu^1,^*, Renchao Che^6,^*

^1^ Key Laboratory of Jiangxi Province for Persistent Pollutants Control and Resources Recycle, School of Environmental and Chemical Engineering, Nanchang Hangkong University, Nanchang 330063, P. R. China

^2^ State Environmental Protection Key Laboratory of Environmental Risk Assessment and Control on Chemical Process, School of Mechanical and Power Engineering, East China University of Science and Technology, Shanghai 200237, P. R. China

^3^ Key Laboratory of Nondestructive Testing, Ministry of Education, School of Instrument Science and Optoelectronic Engineering, Nanchang Hangkong University, Nanchang 330063, P. R. China

^4^ School of Physics and Materials Science, Nanchang University, Nanchang 330031, P. R. China

^5^ College of Chemistry & Materials, Jiangxi Agricultural University, Nanchang 330045, China

^6^ Laboratory of Advanced Materials, Shanghai Key Lab of Molecular Catalysis and Innovative Materials, Academy for Engineering & Technology, Fudan University, Shanghai 200438, P. R. China

^†^Tong Huang, Dan Wang, Xue He, and Zhaobo Feng contributed equally to this work.

*Corresponding authors. Email: [pengyuhui@nchu.edu.cn](mailto:pengyuhui@nchu.edu.cn) (Yuhui Peng); [gsluo566@163.com](mailto:gsluo566@163.com) (Guangsheng Luo); [xuliangnie123@163.com](mailto:xuliangnie123@163.com) (Xueliang Nie); [cbliu2002@163.com](mailto:cbliu2002@163.com) (Chongbo Liu); [rcche@fudan.edu.cn](mailto:rcche@fudan.edu.cn) (Renchao Che)

**S1 Materials**

The main chemicals used are terephthaldicarboxaldehyde (PDA), 1,3,5-tris(4-aminophenyl) benzene (TAPB), N,N-dimethylformamide (DMF), tetrahydrofuran (THF), scandium trifluoromethanesulfonate (Sc(OTf)_3_), FeCl_3_·6H_2_O, NiCl_2_·6H_2_O, ethanol, butanol, methanol, and acetone. All the reagents are provided by Shanghai Macklin Biochemical Technology Co., Ltd. and used without further purification.

**S2 Electromagnetic Measurements**

The electromagnetic (EM) properties of the samples are examined between 2 and 18 GHz using a vector network analyzer (Agilent PNA N5224A). The composites are dispersed into paraffin at a loading ratio of 10 wt.%, and a coaxial ring with an inner diameter of 3.04 mm and an outer diameter of 7 mm is processed using a mold. As an electromagnetic wave absorption (EMWA) material, impedance matching, reflection loss (*RL*) and attenuation constant (*α*) are the important evaluation indexes. According to transmission line theory, *RL* and *α* can be calculated by the following formula:

$Z_{in}=\sqrt{\frac{\mu_{r}}{\varepsilon_{r}}}tanh\left[ j(2\pi fd/c)\sqrt{\mu_{r}\varepsilon_{r}} \right]$ (S1)

$RL=20log\left| {(Z}_{in}-1)/{(Z}_{in}+1) \right|$ (S2)

$\alpha=\frac{\sqrt{2}\pi f}{c}\sqrt{(\mu"\varepsilon"-\mu'\varepsilon')+\sqrt{{(\mu'\varepsilon"+\mu"\varepsilon')}^{2}+{(\mu"\varepsilon"-\mu'\varepsilon')}^{2}}}$ (S3)

where *Z_in_* is the normalized input impedance of EMWA materials, *Z_0_* is the free space impedance; *ε_r_* and *µ_r_* represent complex permittivity and complex permeability, respectively; *d* is the thickness of the EMWA materials; *f* is the frequency of EMWs. The Debye relaxation formula is an important means of studying polarization effect as follows:

${(\varepsilon'-\frac{\varepsilon_{s}+\varepsilon_{\infty}}{2})}^{2}+{(\varepsilon")}^{2}={(\frac{\varepsilon_{s}-\varepsilon_{\infty}}{2})}^{2}$ (S4)

$\varepsilon'=\varepsilon_{\infty}+\frac{\varepsilon_{s}-\varepsilon_{\infty}}{1+\omega^{2}\tau^{2}}$ (S5)

$\varepsilon''=\frac{\varepsilon_{s}-\varepsilon_{\infty}}{1+\omega^{2}\tau^{2}}\omega\tau+\frac{\sigma}{\omega\varepsilon_{0}}=\varepsilon_{p}"+\varepsilon_{c}"$ (S6)

where *ε_∞_* represents the relative dielectric permittivity at infinite frequency and *ε_s_* is the static dielectric permittivity, *ω* is the angular frequency, *τ* is the relaxation time and *σ* is the electric conductivity.

Equation S7 can be derived from Equation S6 in order to extract the relaxation time of each polarization behavior [S1, S2]:

$\varepsilon'=\varepsilon_{\infty}+\frac{1}{\omega\tau}\cdot\varepsilon"$ (S7)

The reflection coefficient (*R*) is calculated as:

$R={|\frac{\sqrt{\mu_{r}/\varepsilon_{r}}-1}{\sqrt{\mu_{r}/\varepsilon_{r}}+1}|}^{2}$ (S8)

*R* < 0.1 (absorptivity > 90%) is a standard threshold for effective EMWA [S3].

**S3 RCS Simulation**

The actual far-field response of the material is simulated using CST Studio Suite 2022. The material is modeled as a two-layer square model with a metallic base plate and an absorber layer, where the size of the square is 200 mm × 200 mm, the thickness of the absorber layer is x mm (x corresponds to the thickness of *RL_min_* for each set of samples), and the thickness of the bottom perfectly conducting layer (PEC) is set to 2.0 mm. The model is placed in the X-O-Y plane, and the EMWs are incident from the positive direction of Z-axis. Adjusting the incident angle θ (−90°-90°) of EMWs while keeping the polarization angle φ constant, the RCS value can be expressed by *S* (area of the model), *λ* (wavelength of the EMW), *𝐸_𝑠_* (electric field intensity of the scattered wave) and *𝐸_𝑖_* (electric field intensity of incident wave) [S4, S5].

$RCS\left( {dBm}^{2} \right)=10\log\left( \left( \frac{4\pi S}{\lambda^{2}} \right)\left| \frac{E_{s}}{E_{i}} \right| \right)^{2}$ (S9)

**S4 DFT calculation**

The Density Functional Theory (DFT) calculations are carried out with the Vienna Ab initio Simulation Package (VASP) code [S6]. The Perdew-Burke-Ernzerhof (PBE) [S7] functional within generalized gradient approximation (GGA) is used to process the exchange-correlation, while the projector augmented-wave pseudopotential (PAW) [S8, S9] is applied with a kinetic energy cut-off of 550 eV, which is utilized to describe the expansion of the electronic eigenfunctions. The vacuum thickness is set to 15 Å to minimize interlayer interactions. The Brillouin-zone integration is sampled by a Monkhorst-Pack k-point. All atomic positions are fully relaxed until energy and force reaches a tolerance of 1 × 10^−5^ eV and 0.01 eV/Å, respectively.

**S5 Finite element simulation**

To analyze the variation of EM response characteristics, COMSOL Multiphysics 6.2 is utilized to simulate the distributions of the electric field and power loss density for the typical samples. The samples are placed in the middle (Part 1) for simulation, and Part 2 is determined as the free space. To meet the real environment, Part 3 is set as the perfect matching layer (PML) and the other boundary conditions are all modified to periodic boundary conditions (PBC). In addition, two ports are also set at the interfaces of free space and PML, wherein the top port is the EM waves excitation signal with a transport type of periodicity and the bottom port is aimed to receive the transmitted EM wave. In addition, ascribing to the scale difference between the model and EM wavelength, the dimension absorbing model is enlarged by suitable times and obtained the numerical solution [S10].

**S6 Micromagnetic Simulation**

The micromagnetic simulation is carried out by Mumax3 software, which is an opensource software based on graphics card acceleration. According to the Landau-Lifshitz-Gilbert equation [S11] and the principle of minimum energy as well as the finite difference algorithm, the dynamic physical process simulation are carried out. In this work, the simulation is carried out to describe dynamic spin structure of magnetic nanoparticle microspheres with different spacings. The following magnetic parameters are used during the simulation: the value of saturation magnetization for is 4.9 × 10^5^ A/m, The micromagnetic exchange constant is 9×10^-12^ J/m. Besides, the Landau-Lifshitz damping constant is 0.01. The frequency of applied magnetic field in the simulation is 5.2 GHz.

**Supplementary Figures and Table**

A schematic of the preparation method of NF@NCA composites is illustrated in **Figure S1**. Sc(OTf)_3_ with high catalytic activity plays a prominent role by participating in the hydrogen bonding between the amine and aldehyde groups. Under the action of the Sc(OTf)_3_ catalyst, the monomers containing amine and aldehyde groups initiate the formation of small sol-gel particles through aldimine condensation reaction. The sol-gel particles link together into chains that expand into high-dimensional networks and coalesce via bottom-up approach to form crystalline gels in a relatively short time. Further, the solvent exchange and metal salt impregnation processes ensure even distribution of metal ions in the gel through coordination bonds, which stabilizes the gel network. The resulting NF@N-doped carbon aerogel (NCA) is prepared through freeze drying and high-temperature thermal reduction.


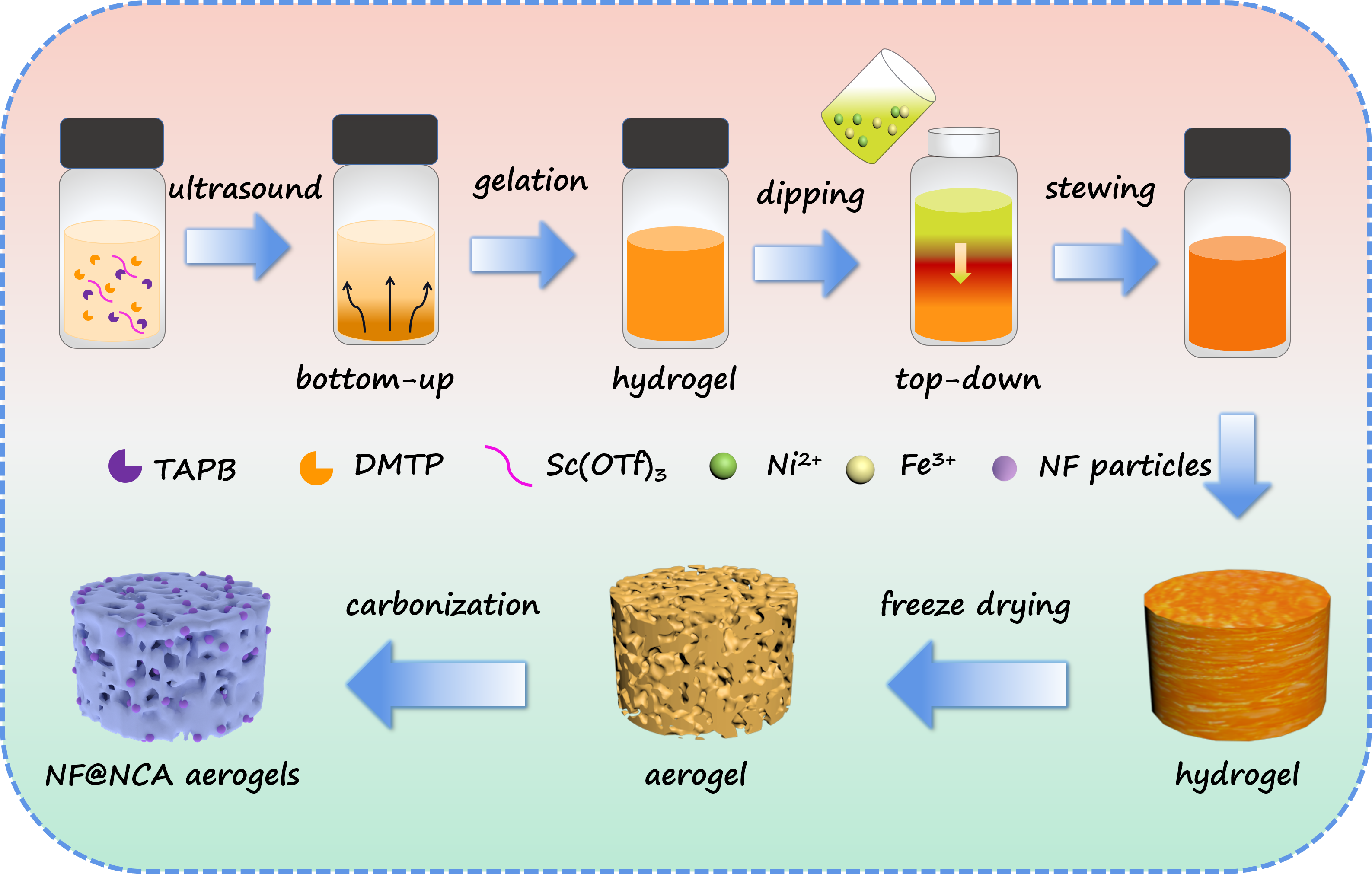


**Fig. S1** Schematic of the preparation method of NF@NCA composites

The XRD pattern of this NCA precursor displays four characteristic patterns with intensely peak at 2.7°, 4.8°, 5.6° and 7.4°, respectively (**Fig. S2a**). NCA precursor transforms into porous carbon. As shown in **Fig. S2b**, the strongest diffraction peak can be attributed to amorphous carbon.


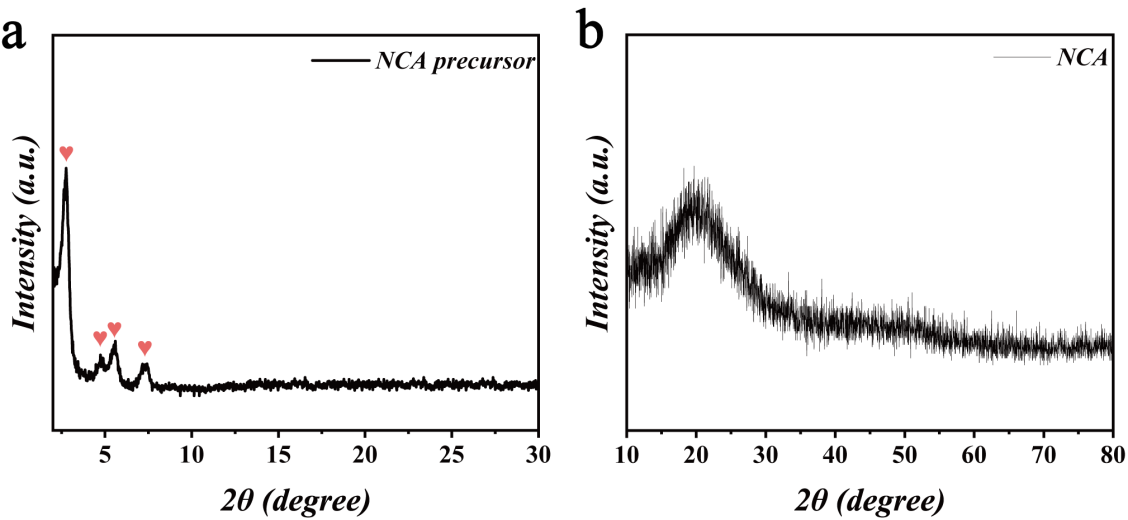


**Fig. S2** XRD pattern for (**a**) NCA precursor and (**b**) NCA

The chemical bonds of the sample are characterized using FT-IR spectroscopy. As shown in **Fig. S3a-b**, C-C and C=N stretching vibration peaks appear near 1596 cm^-1^ as well as 1621 cm^-1^, which indicates the successful synthesis of NCA precursor. With the addition of metal ions (Fe^3+^ and Co^2+^), the strength of C-C and C=N stretching vibration peaks obviously weaken and shift to the high wavenumber, proving the successful formation of stable coordination bonds between Fe/Co and N.


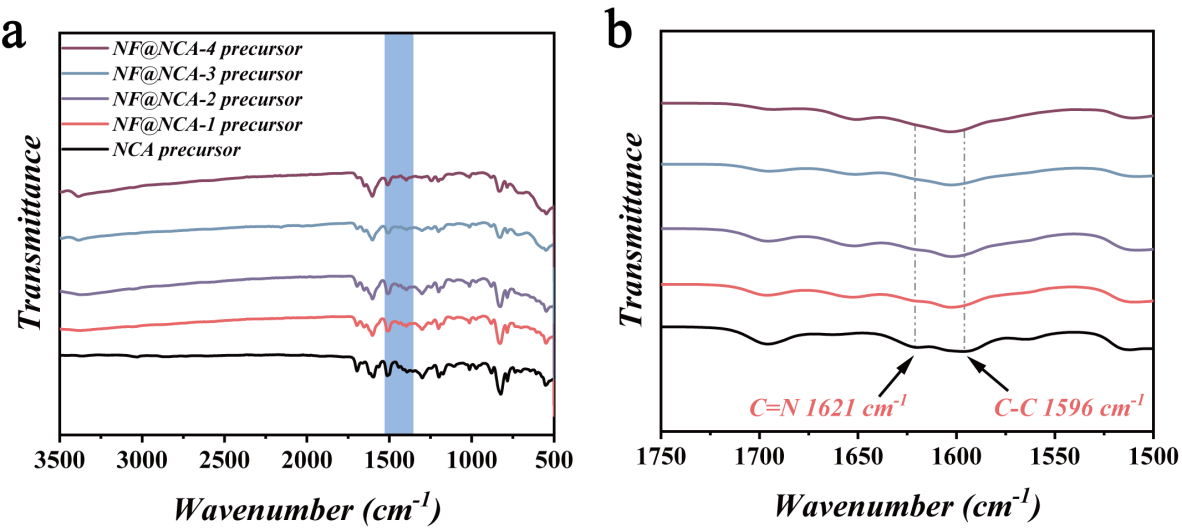


**Fig. S3** FT-IR for (**a**) NCA, NF@NCA-1, NF@NCA-2, NF@NCA-3, NF@NCA-4 precursors and (**b**) enlarged image


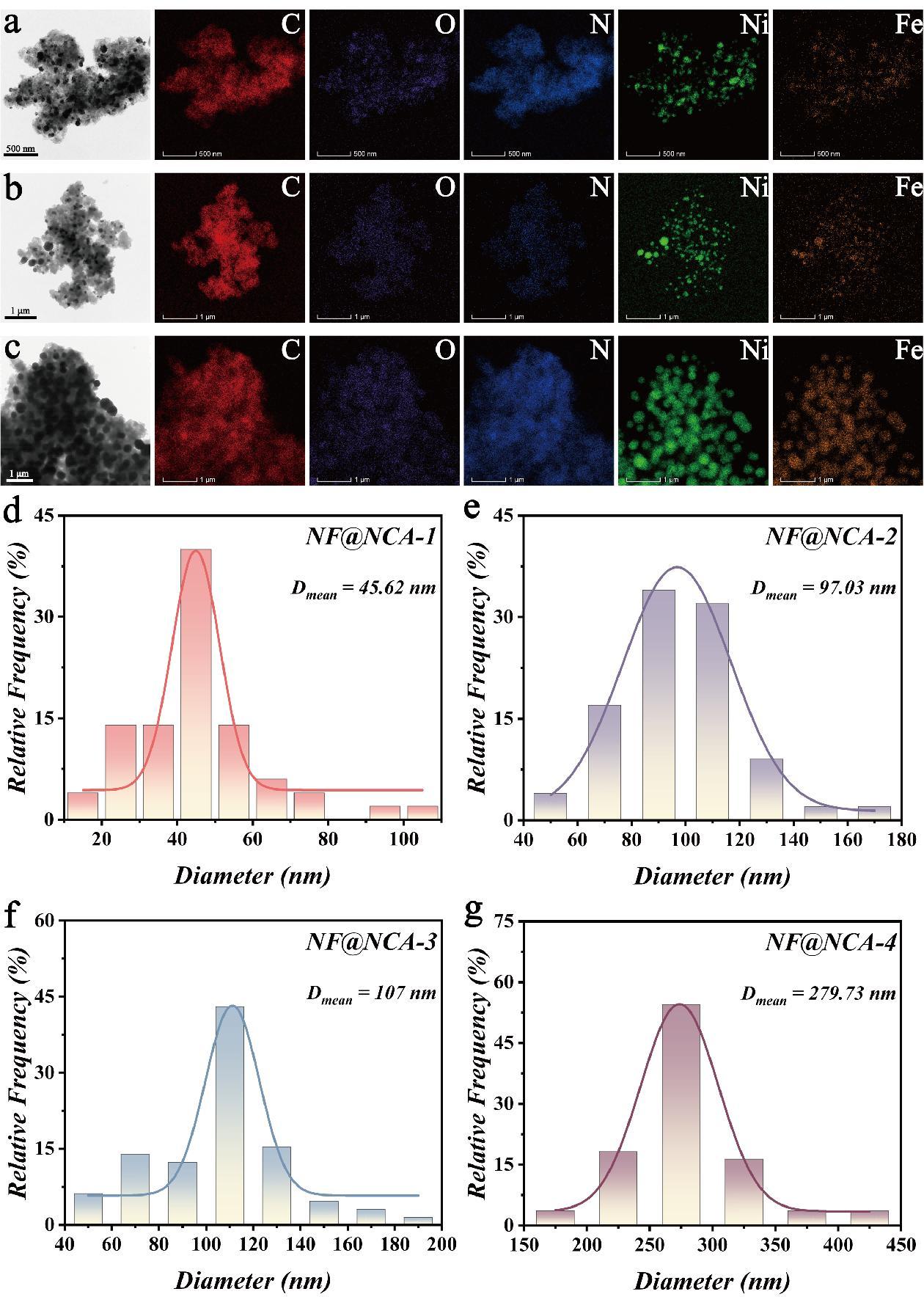


**Fig. S4** EDS mapping for (**a**) NF@NCA-1, (**b**) NF@NCA-2, and (**c**) NF@NCA-4. Particle size distributions for (**d**) NF@NCA-1, (**e**) NF@NCA-2, (**f**) NF@NCA-3, and (**g**) NF@NCA-4

**
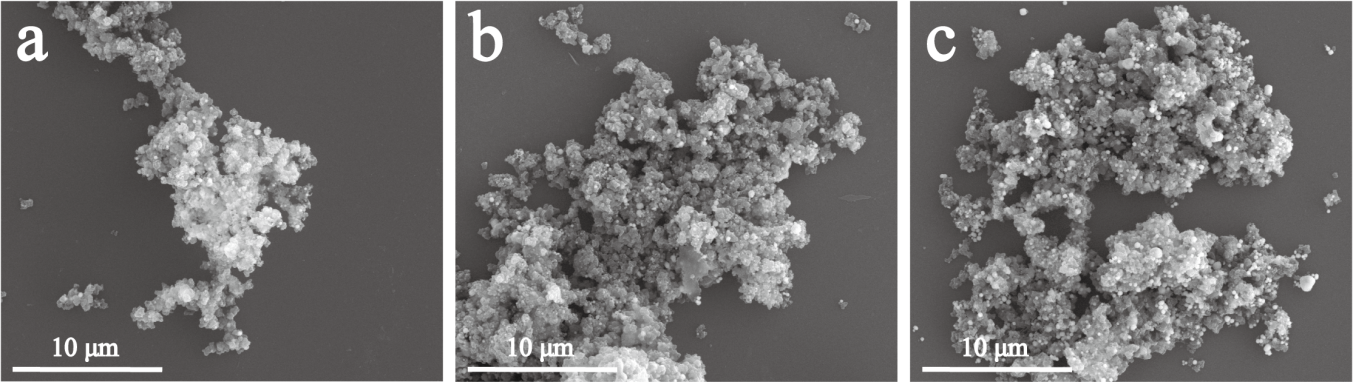
**

**Fig. S5** SEM images of (**a**) NF@NCA-1, (**b**) NF@NCA-2, and (**c**) NF@NCA-4

**Figure S6a** shows the typical type-IV curve characteristics as well as the BET surface area of the NF@NCA composites, which decreases from 464.8 m^2^/g to 324.87, 309.7, and 244.5 m^2^/g with the increase in metal salt concentration. At the same time, the pore size distribution curves reveal the coexistence of micropores and mesopores in the NF@NCA composites. The BJH curves indicate that the average pore diameter of all NF@NCA composites is less than 4.0 nm. Specifically, the pore volumes of NF@NCA composites are 0.33, 0.29, 0.25, and 0.22 cm^3^/g (**Fig. S6b**). According to the above analysis, it can be inferred that NCA composites have a large specific surface area and high porosity. However, the introduction of excessive NF nanoparticles can affect the specific surface area and pore structure of the aerogel.


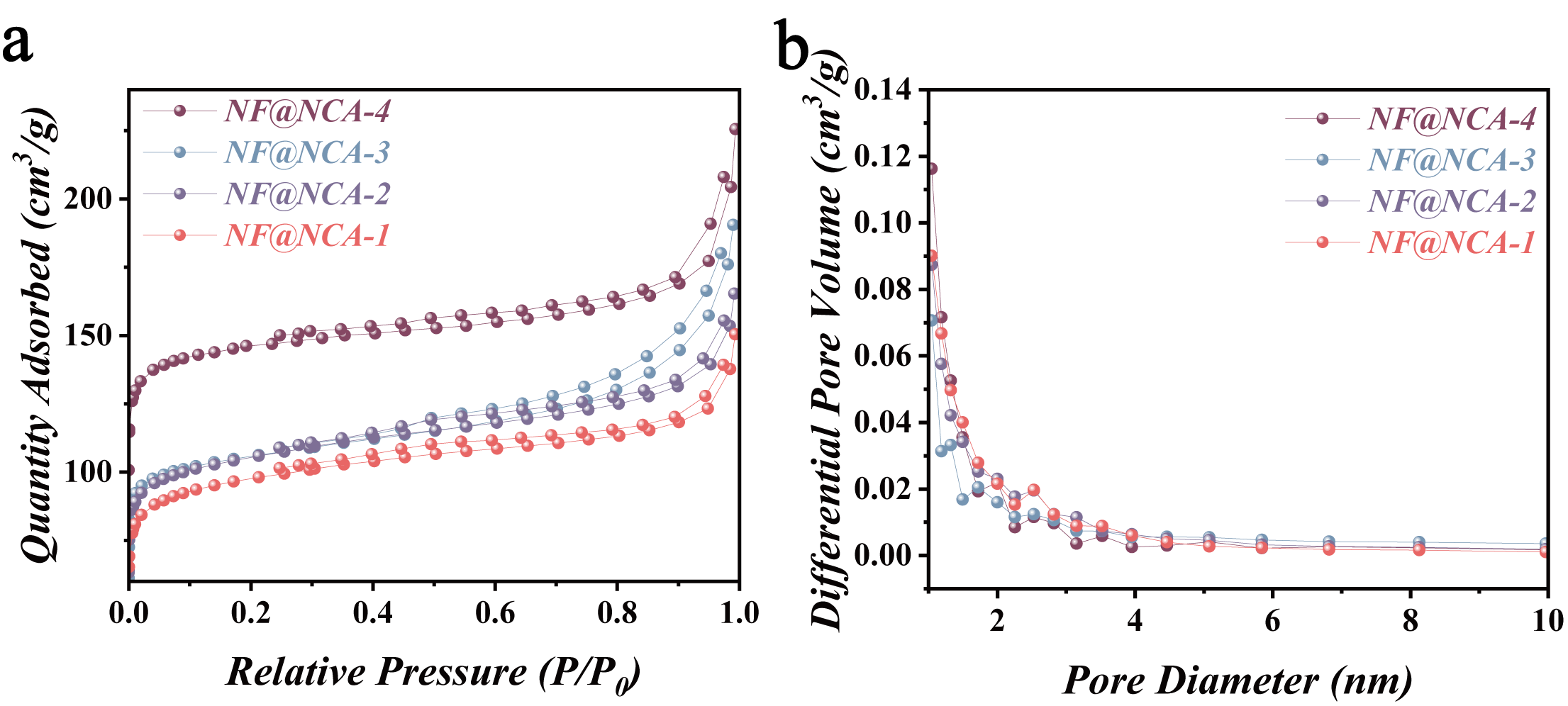


**Fig. S6** (**a**) N_2_ adsorption-desorption isotherms and (**b**) pore size distribution for NF@NCA composites

To determine the near-surface elements and their valence states, the NF@NCA-3 composites were examined by XPS. The survey spectrum reveals the presence of C, N, O, Ni, and Fe elements in NF@NCA-3 (**Fig. S7a**). The narrow high-resolution spectrum of C 1s shows peaks at 284.8, 286.5, and 289.5 eV, indicating the existence of C-C/C=C, C-N and C-O bonds, respectively (**Fig. S7b**). The N 1s spectrum can be decomposed into three peaks at 398.4, 400.8, and 401.8 eV, which correspond to pyridinic N, pyrrolic N, and graphitic N, respectively (**Fig. S7c**). These results verify that the nitrogen atoms are successfully doped into the carbon lattice in the NF@NCA composites after pyrolysis.


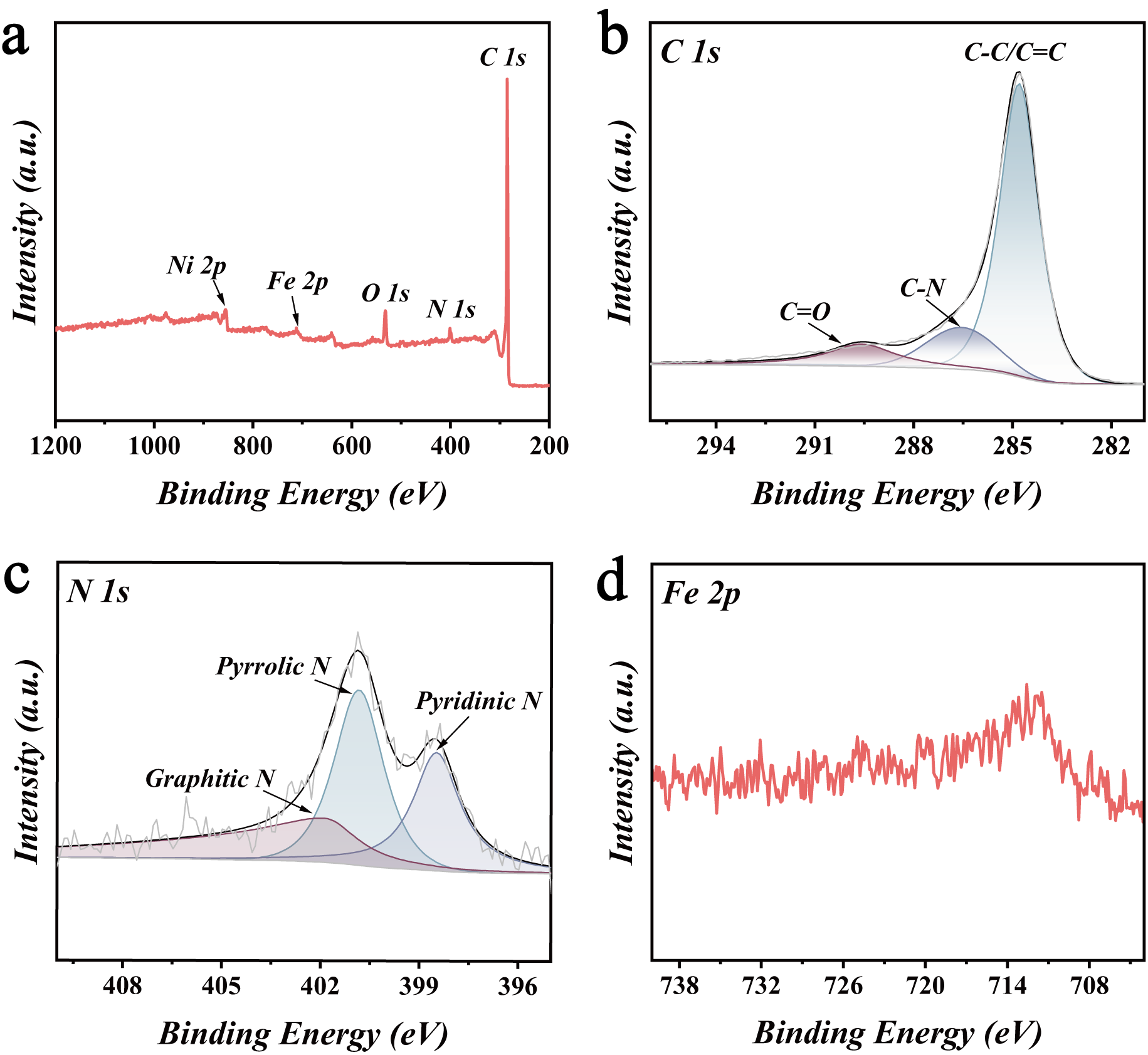


**Fig. S7** (**a**) Survey spectra, (**b**) C 1s, (**c**) N 1s, and (**d**) Fe 2p for NF@NCA-3


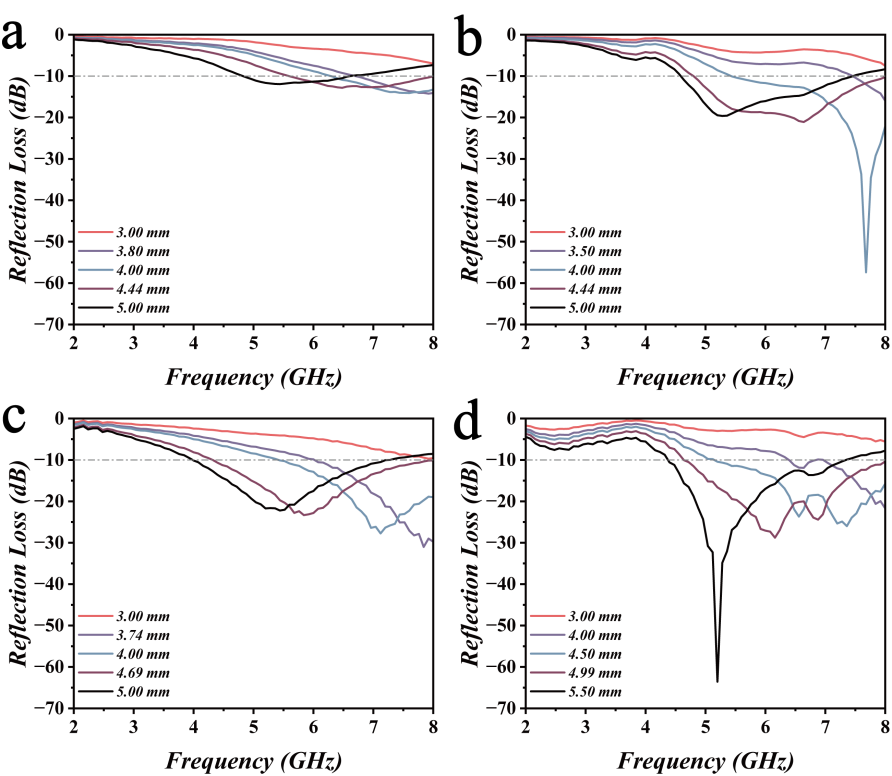


**Fig. S8** EMWA performances for (**a**) NF@NCA-1, (**b**) NF@NCA-2, (**c**) NF@NCA-3, (**d**) NF@NCA-4, respectively

The 3D *RL_min_* mapping of NCA is −24.35 dB at 2.16 GHz, which has the potential application in low-frequency range (**Fig. S9**).


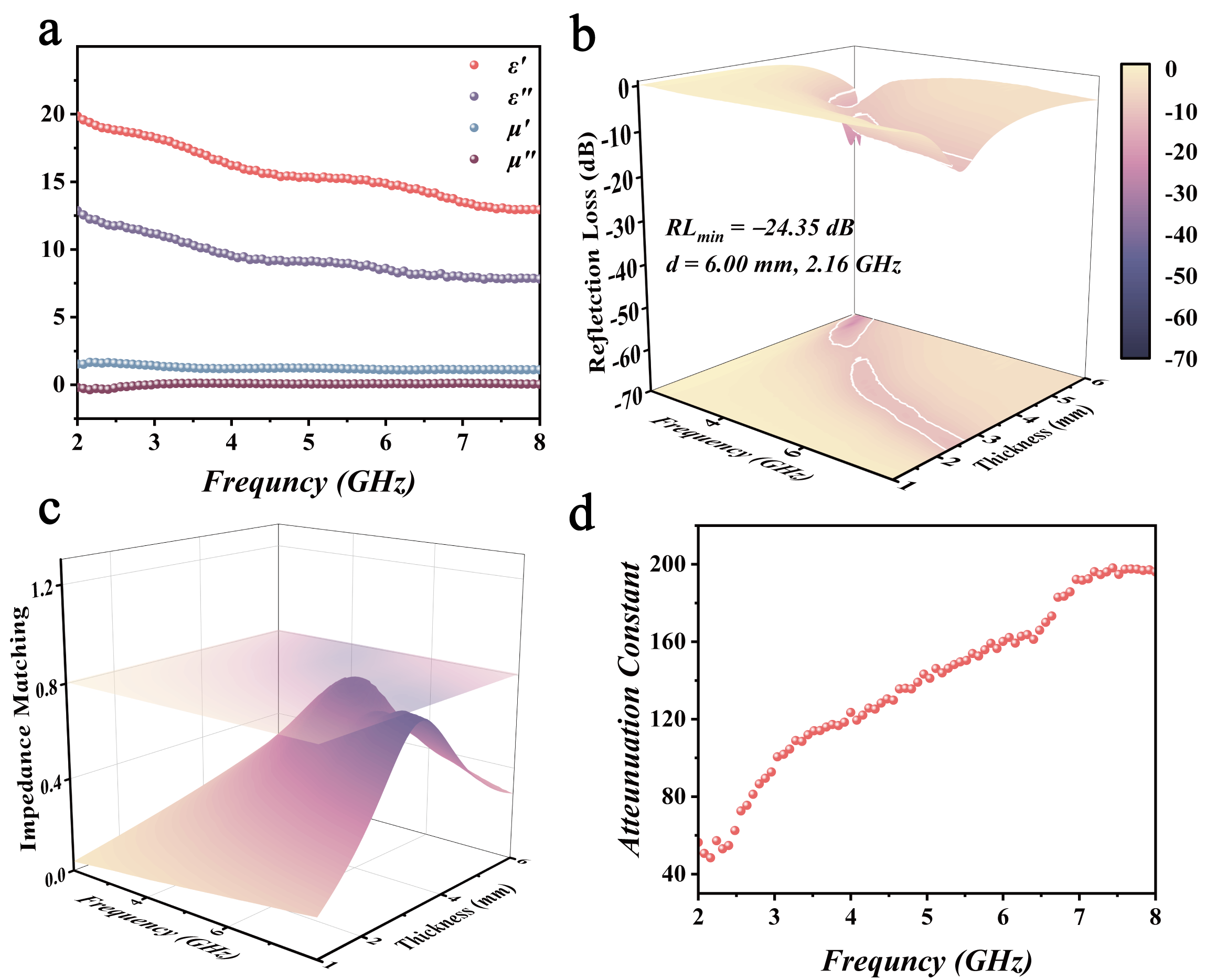


**Fig. S9** (**a**) EM parameters, (**b**) 3D *RL* mapping, (**c**) 3D impedance matching mapping, and (**d**) attenuation constant for NCA


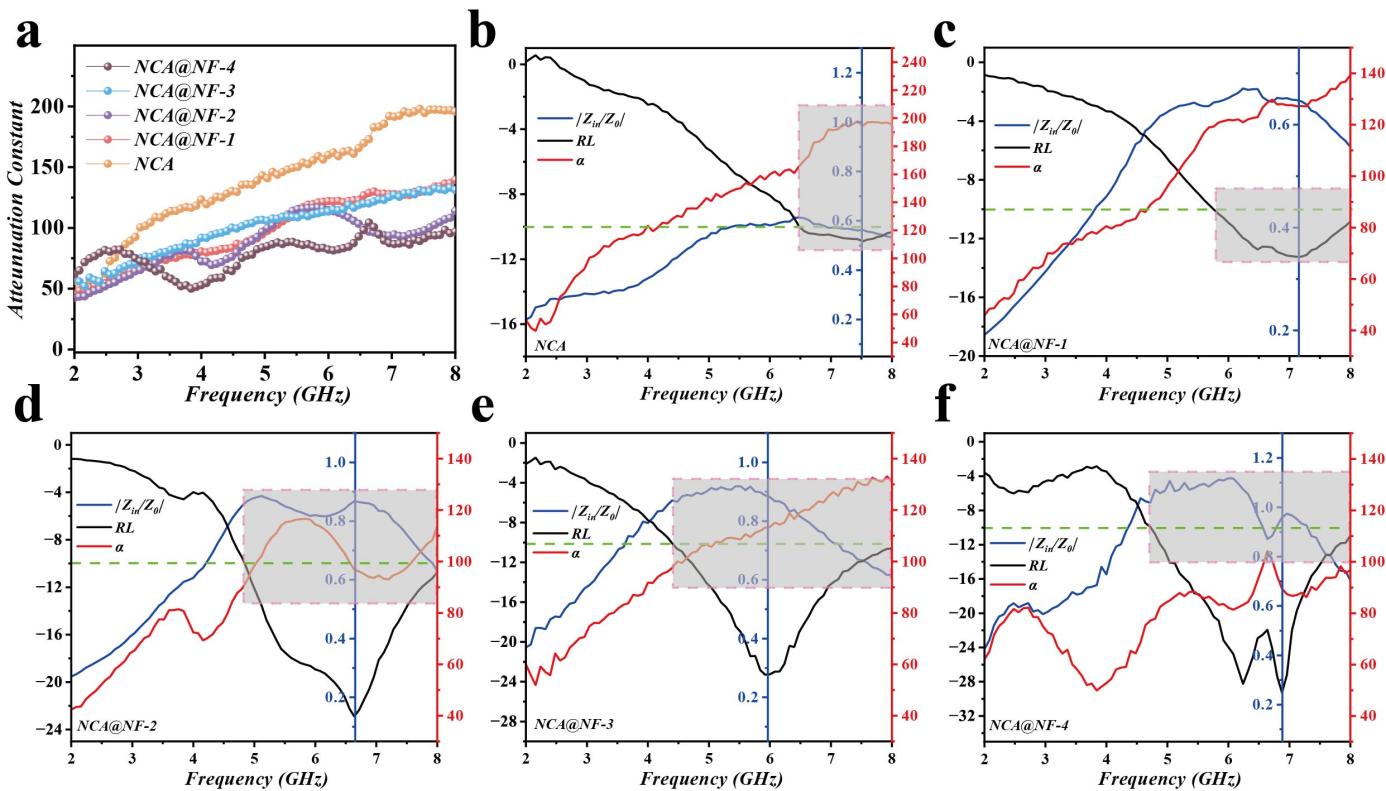


**Fig**. S10 (**a)** *α* values of NCA@NF. Frequency dependence of the |*Z_in_/Z_0_*| values, *α* and *RL* values of (**b)** NCA at 2.7 mm, (**c**) NCA@NF-1 at 4.4 mm, (**d)** NCA@NF-2 at 4.7 mm, (e) NCA@NF-3 at 4.7 mm, (f) NCA@NF-4 at 5.0 mm

The S-W approximation is a way generally used for determining the important parameters such as saturation magnetization or magnetocrystalline anisotropy constant for ferromagnetic materials. It is defined initially as [S12]:

$M=M_{s}(1-\frac{a}{H}-\frac{b}{H^{2}})+\chi_{p}H$ (S10)

*M_s_* is the saturation magnetization, a is the constants depends on inhomogeneity of samples that disappears at high magnetic fields, and *χ_p_* is the high-field differential susceptibility which is active in the high temperature analysis. Hence, the *a*/*H* and *χ_p_H* terms can be neglected and the above equation can reduced to:

$M=M_{s}(1-\frac{b}{H^{2}})$ (S11)

The experimental data of magnetization (*M*) versus applied magnetic field (*1*/*H^2^*) becomes linear when *1*/*H^2^* approaches zero and can be fitted into the positive proportional function above. The plots of M versus *1*/*H^2^* for different samples are indicated in **Fig. S11a**, the slope and intercept of the fitted lines refer to the values of "−*M_S_b*" and "*M_S_*" respectively.

**
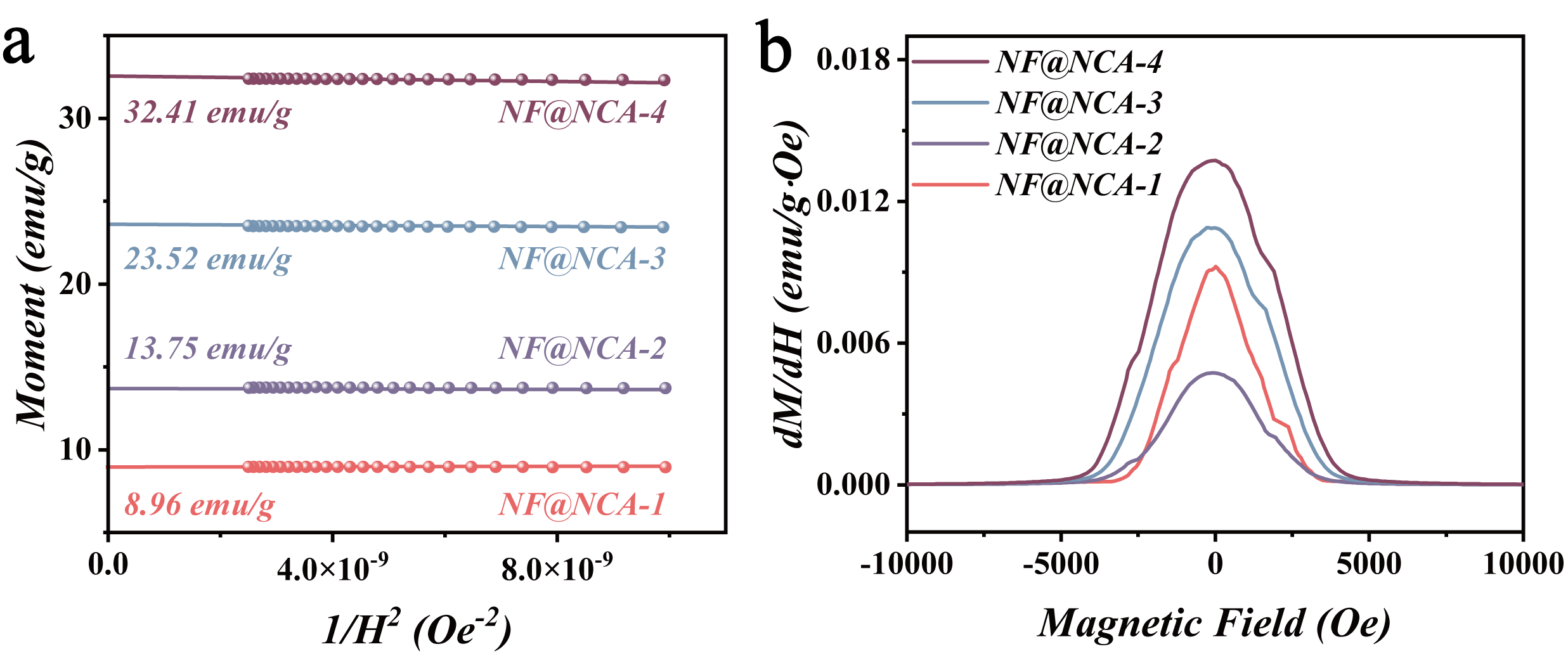
**

**Fig. S11** (**a**) M-*1*/*H^2^* plots and (**b**) switching field distribution curves for NF@NCA composites

EMWA materials are known to convert EM energy into heat or other forms of energy. When EMWs penetrate the material, the total energy (*E_T_*) divides into two components: the storage part (*E_S_*) involving the permittivity and permeability and the dissipation part (*E_D_*) involving the permittivity and permeability, the formula is as follows [S13, S14]:

$E_{T}=E_{S}+E_{D}$ (S12)

*E_S_* is contributed by the real part of the permittivity and permeability, which can be explained as follows:

$E_{S}=\pi f\varepsilon'E_{0}^{2}+\pi f\mu'H_{0}^{2}$ (S13)

*E_D_* is contributed by the imaginary part of the permittivity and permeability, which can be expressed as follows:

$E_{D}=\pi f\varepsilon"E_{0}^{2}+\pi f\mu"H_{0}^{2}$ (S14)

where *E_0_* and *H_0_* are the electric and magnetic field intensity amplitude of EMWs, respectively.

As the loss can be divided into polarization (*ε_p_"*), conduction (*ε_c_"*), and magnetic loss (*µ"*):

$E_{P}=\pi f{\varepsilon_{p}"E}_{0}^{2}$ (S15)

$E_{C}=\pi f{\varepsilon_{c}"E}_{0}^{2}$ (S16)

$E_{M}=\pi f{\mu"H}_{0}^{2}$ (S17)

where *E_P_*, *E_C_*, *E_M_* represent the polarization loss, conduction loss, and magnetic loss, respectively.

The polarization loss contribution (*R_P_*) and conduction loss contribution (*R_C_*), and magnetic loss contribution (*R_M_*) can be expressed as follows:

$R_{P}=\frac{E_{P}}{E_{D}}$ (S18)

$R_{C}=\frac{E_{C}}{E_{D}}$ (S19)

$R_{M}=\frac{E_{M}}{E_{D}}$ (S20)

The results can be calculated using "https://github.com/fbpuf/energy-conversion-analysis".

The equivalent circuit model includes a heat dissipation module at the bottom and a conductive module at the top. In the conduction module, similar P-type and N-type semi-conductors in the material generate space charges with opposite potentials, creating a built-in electric field. As the EMWs propagate into the composite, hole and electron pairs are excited in the semiconductors. Under the influence of the built-in electric field, the excited electrons migrate directionally toward the conduction module, generating microcurrents. During carrier migration, a portion of the EMW energy is converted into thermal energy due to electron relaxation polarization, lattice scattering, and hysteresis effects. And then the heat energy is transferred to the metal substrate below. A temperature difference is formed between the metal substrate as a heat source and collectors as the cold source. Based on the Seebeck effect, the temperature difference current is formed in the heat conversion module. The collectors at both ends of the cell device integrate the two currents to form a total current, powering the external circuit equipment. Furthermore, the metal substrate can reflect EMWs, which increases the propagation route of EMWs in composites, and improves the absorption efficiency for EM energy (**Fig. S12**).


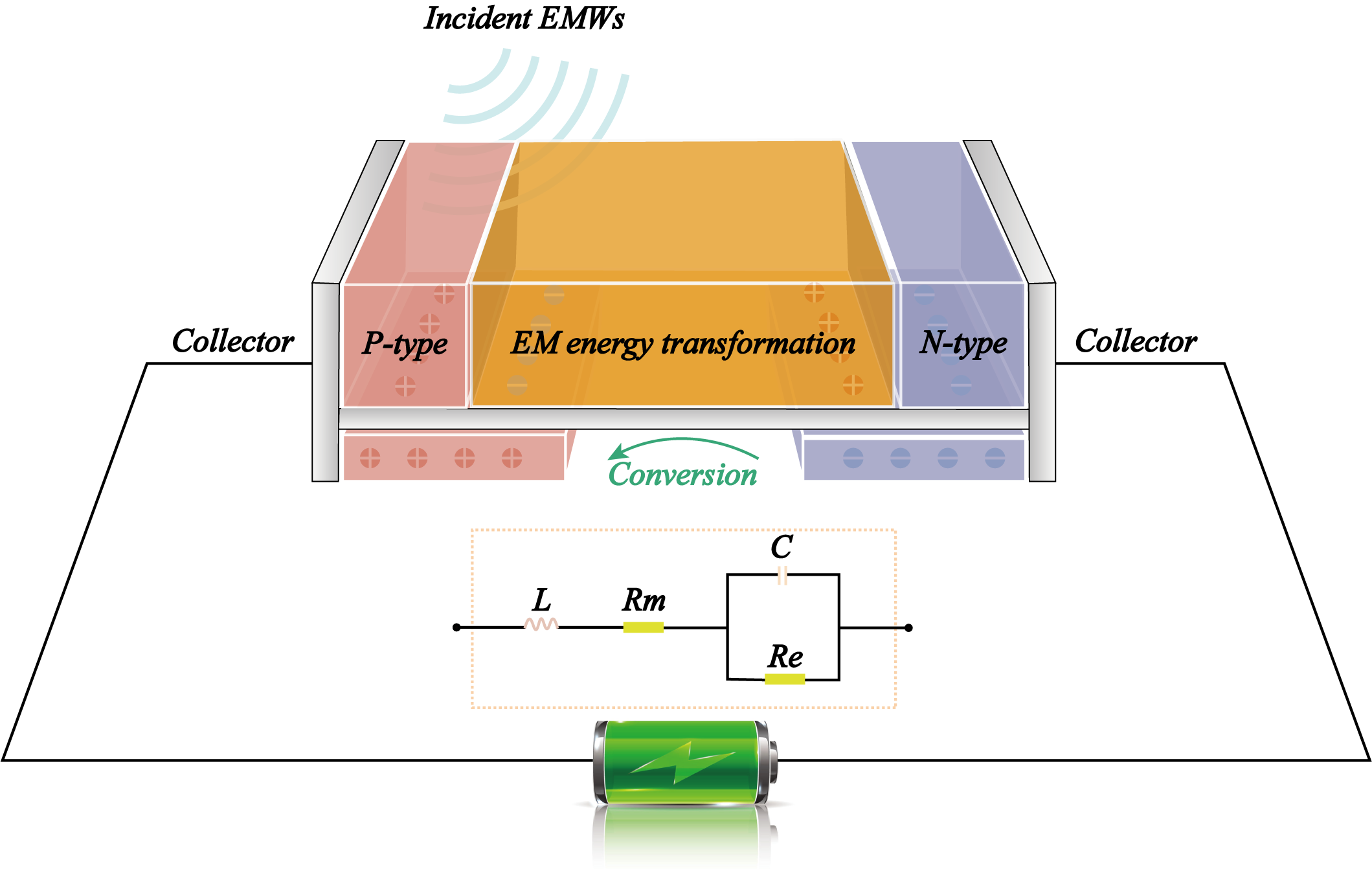


**Fig. S12** Schematic diagram of energy conversion device and equivalent circuit model. The device consists of a conductive module (upper) and a thermal conversion module (lower)

**
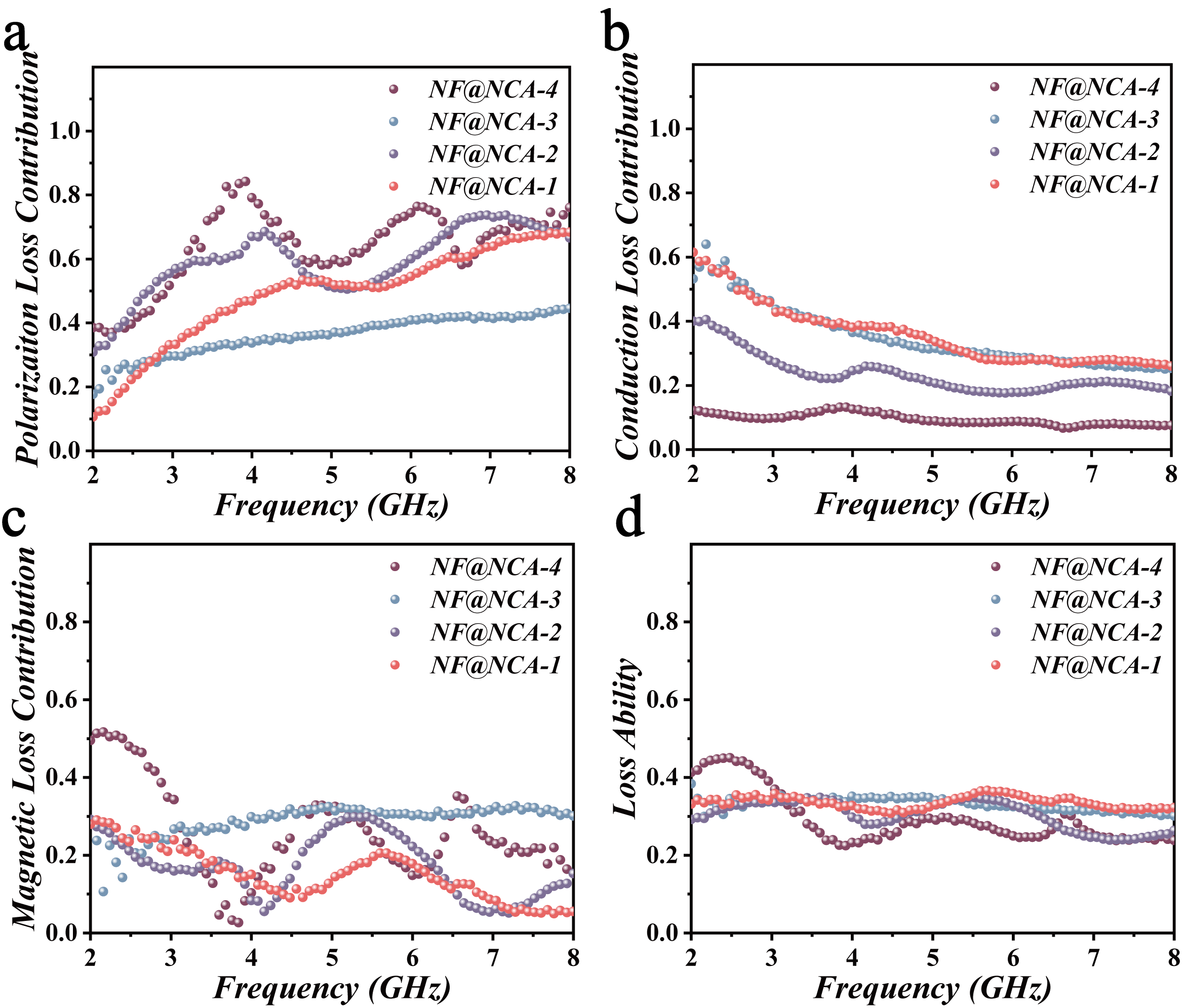
**

**Fig. S13** (**a**) Polarization loss contribution, (**b**) conduction loss polarization, (**c**) magnetic loss contribution, and (**d**) loss ability for NF@NCA composites

**
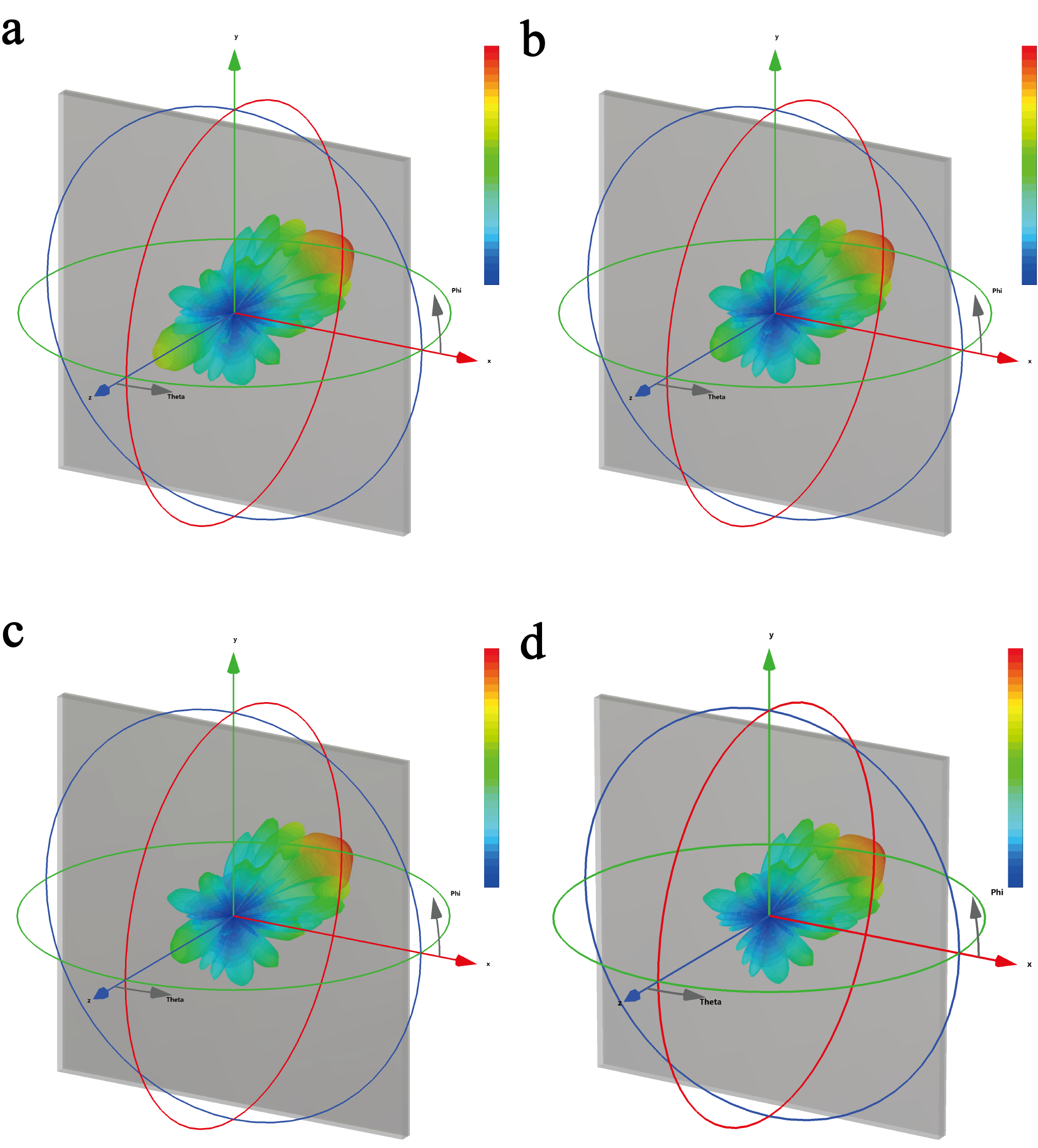
**

**Fig. S14** 3D RCS plots for (**a**) NF@NCA-1, (**b**) NF@NCA-2, (**c**) NF@NCA-3, and (**d**) NF@NCA-4

The actual thickness of the six samples is matched to the simulated thickness (Equation S21). With an increase in thickness, the absorption peaks of the *RL* gradually shift to lower frequencies (**Fig. S15**).

The relation between the *RL*, thickness, and frequency can be obtained using the quarter-wavelength model. The simulated thickness (*t_m_*) of an absorber at 2–8 GHz is estimated as follows:

$t_{m}=nc/(4f_{m}\sqrt{|\mu_{r}||\varepsilon_{r}|})$ (S21)

where t_m_, n, and f_m_ denote the simulated thickness, the refractive index of the material, and the matched frequency, respectively.

**
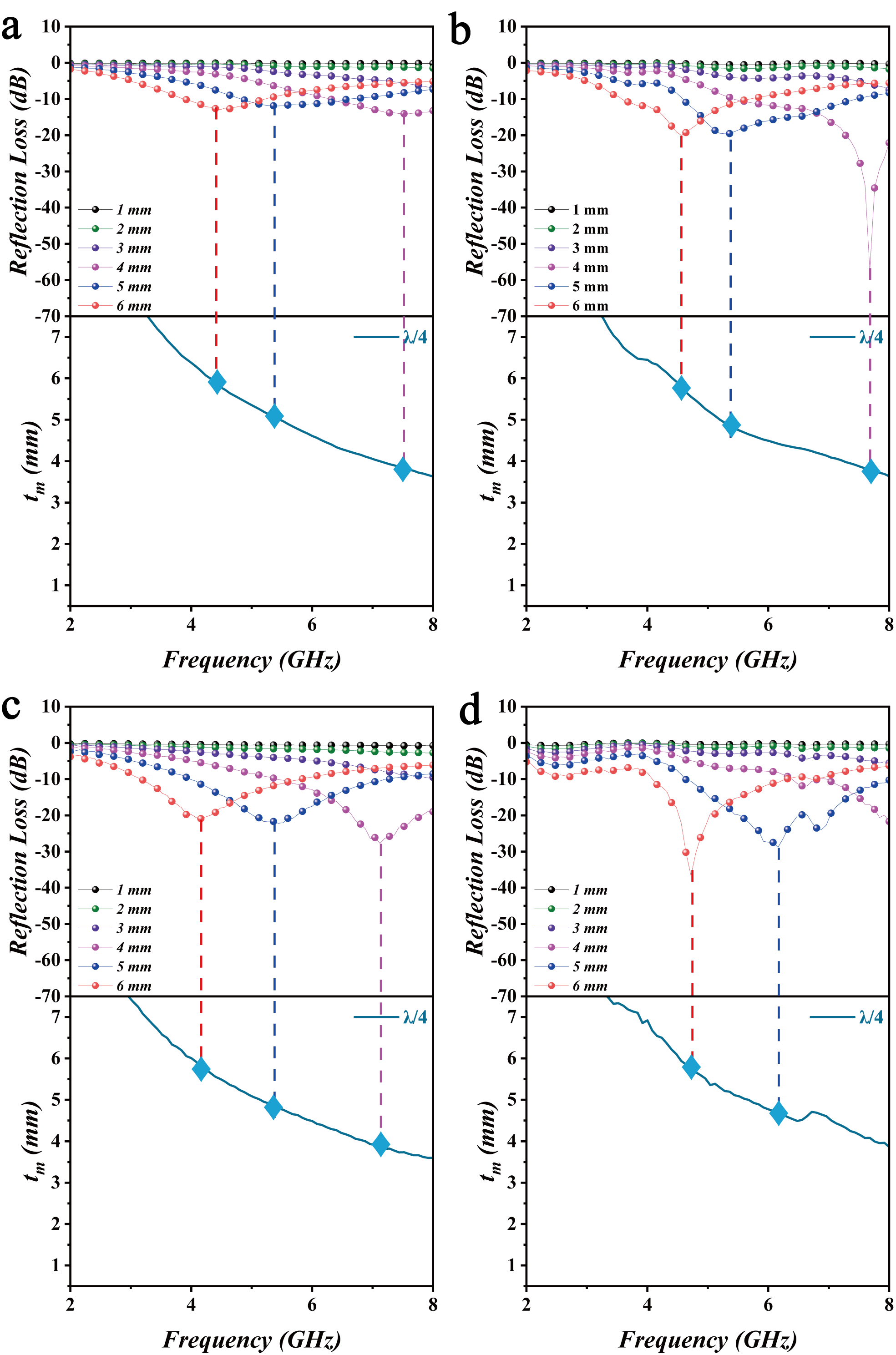
**

**Fig. S15** The relation between the *RL*, thickness, and frequency for (**a**) NF@NCA-1, (**b**) NF@NCA-2, (**c**) NF@NCA-3, and (**d**)NF@NCA-4

Based on the permittivity and permeability of NF@NCA-3, we have calculated the reflection loss curves of the multilayered metamaterials with different geometry parameters by CST Studio Suite 2022 [S16]. Floquet boundary conditions are used in this process, the frequency range is set to 2-40 GHz, where complex permittivity in the range of 18-40 GHz are fitted by the Cole-Cole model [S17], and the structure is meshed in a tetrahedral form. The overall unit cell is defined by 11 parameters: seven horizontal parameters (L1–L7) and four vertical parameters (H1–H4). The optimized metamaterial design is shown in **Fig. S16l**. The dimensions for L1, L2, L3, L4, L5, L6, L7, H1, H2, H3, and H4 are 27, 25, 24, 22, 21, 18, 3, 4, 4, 5, and 1mm, respectively.


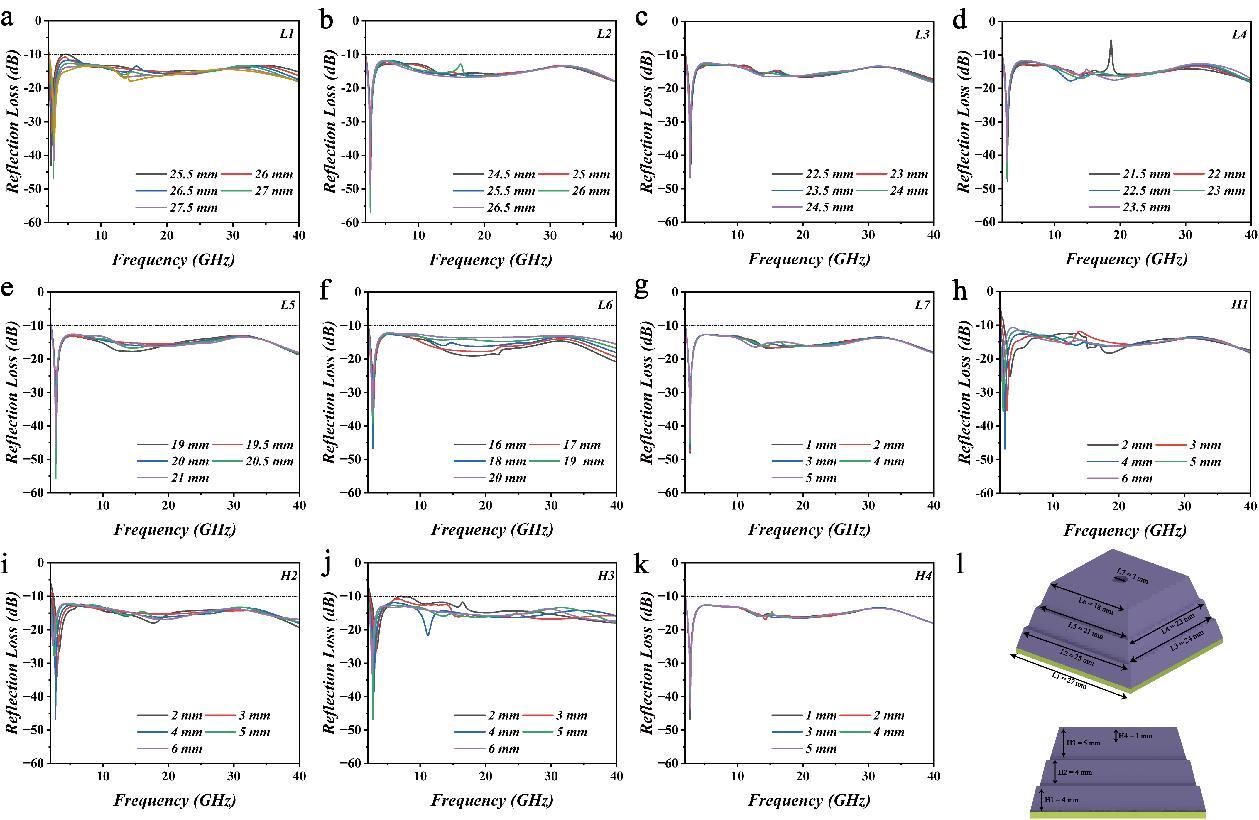


**Fig. S16** By changing (**a**) L1, (**b**) L2, (**c**) L3, (**d**) L4, (**e**) L5, (**f**) L6, (**g**) L7, (**h**) H1, (**i**) H2, (**j**) H3, and (**k**) H4 optimize the reflectivity in the frequency range from 2 to 40 GHz to determine the unit size of the absorber. (**l**) Optimized metamaterial design

**
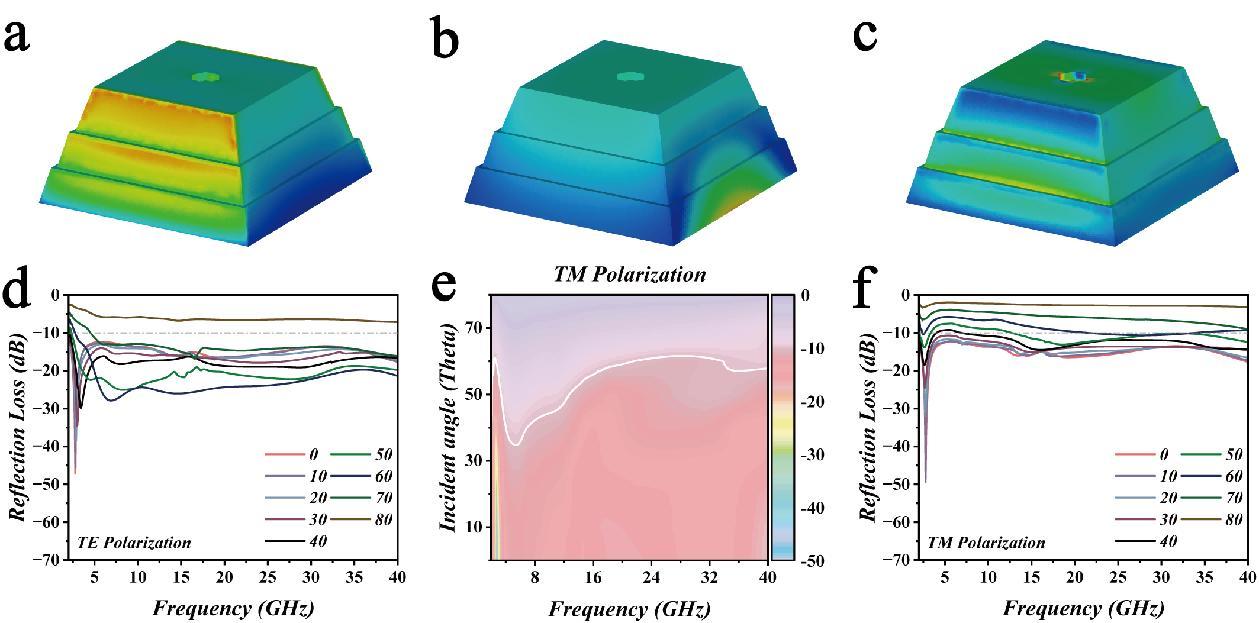
**

**Fig. S17** (**a**) Electric field distribution, (**b**) magnetic field distribution, and (**c**) power loss density for metamaterial at 2.76 GHz. (**d**) Different incident angles analysis for metamaterial under TE polarization mode. (**e-f**) Different incident angles analysis for metamaterial under TM polarization mode


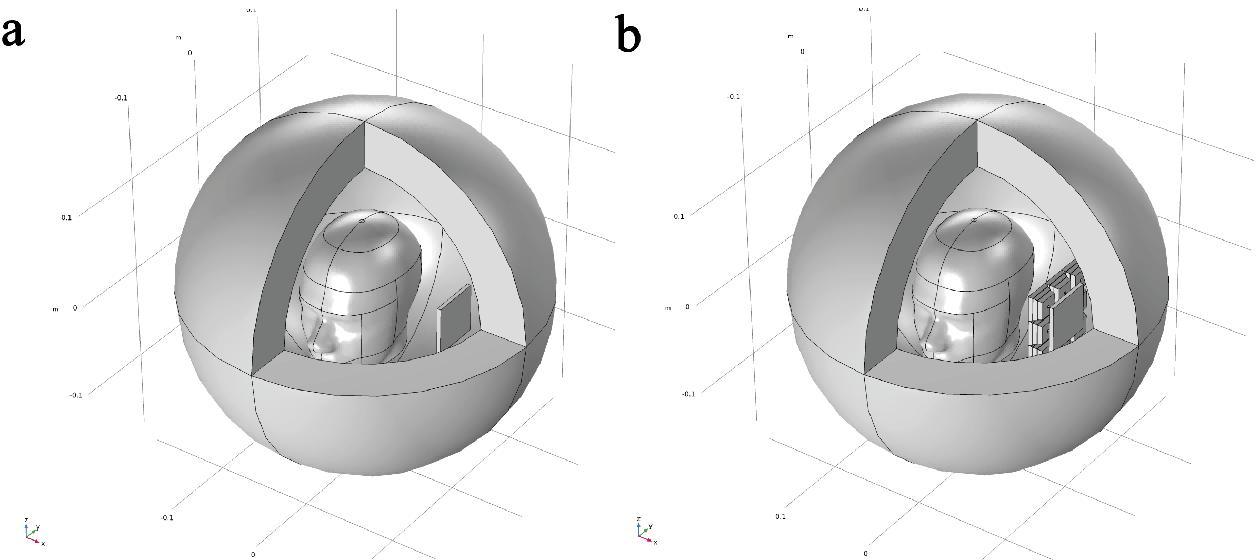


**Fig. S18** (**a**) The SAR model of PML, antenna, and brain. (**b**) The SAR model coated with metamaterial

**
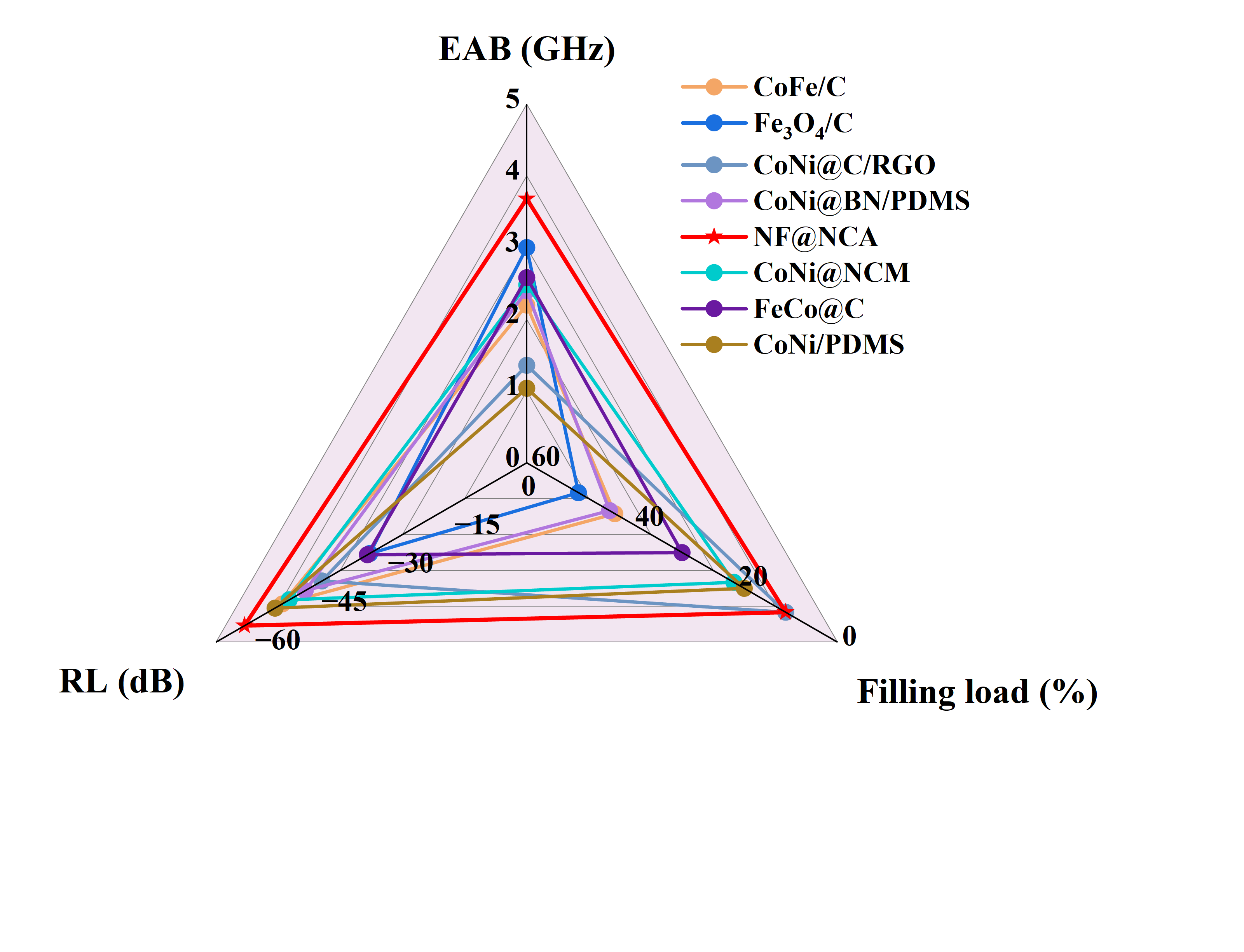
**

**Fig. S19** Low-frequency performance comparison (2-8 GHz)

**Table S1** Low-frequency performance comparison (2-8 GHz)

| **Filler** | ***RL_min_* [dB]** | **EAB [GHz]** | **Filling ratio** | **Refs.** |
| --- | --- | --- | --- | --- |
| CoFe/C | −55.05 | 2.2 (5.4-7.6) | 43% | [S17] |
| Fe_3_O_4_/C | −35.40 | 3 (~4-7) | 50% | [S18] |
| CoNi@C/RGO | −46.14 | 1.36 (4.79-6.15) | 10% | [S19] |
| CoNi@BN/PDMS | −49.90 | 2.4 (3.92-6.32) | 44% | [S20] |
| **NF@NCA** | **−63.57** | **3.68 (4.32-8)** | **10%** | **This work** |
| CoNi@NCM | −53.50 | 2.48 | 20% | [S21] |
| FeCo@C | −35.90 | 2.58 (5.20-7.78) | 30% | [S22] |
| CoNi/PDMS | −56.70 | 1.04 (2.96-4) | 18% | [S23] |

**Supplementary References**

1. L. Li, F. Pan, H. Guo, H. Jiang, X. Wang et al., Tailored magnetic spatial confinement with enhanced polarization and magnetic response for electromagnetic wave absorption. Small **20**(46), 2402564 (2024). <https://doi.org/10.1002/smll.202402564>
2. J. Lin, Y. Peng, J. Luo, Z. Xiong, J. Huang et al., One-click to 3D: helical carbon nanotube-mediated MXene hierarchical aerogel with layer spacing engineering for broadband electromagnetic wave absorption. Small Meth. **9**(5), 2401665 (2025). <https://doi.org/10.1002/smtd.202401665>
3. J. Tao, J. Zhou, Z. Yao, J. Wang, L. Xu et al., Multi-field coupled motion induces electromagnetic wave absorbing property regeneration of elastomer in marine environment. Adv. Funct. Mater. **34**(12), 2310640 (2024). <https://doi.org/10.1002/adfm.202310640>
4. W. Huang, M. Song, S. Wang, B. Wang, J. Ma et al., Dual-step redox engineering of 2D CoNi-alloy embedded B, N-doped carbon layers toward tunable electromagnetic wave absorption and light-weight infrared stealth heat insulation devices. Adv. Mater. **36**(30), 2403322 (2024). <https://doi.org/10.1002/adma.202403322>
5. X. Li, R. Hu, Z. Xiong, D. Wang, Z. Zhang et al., Metal-organic gel leading to customized magnetic-coupling engineering in carbon aerogels for excellent radar stealth and thermal insulation performances. Nano-Micro Lett. **16**(1), 42 (2023). <https://doi.org/10.1007/s40820-023-01255-7>
6. G. Kresse, J. Furthmüller, Efficient iterative schemes for *ab initio* total-energy calculations using a plane-wave basis set. Phys. Rev. B **54**(16), 11169–11186 (1996). <https://doi.org/10.1103/physrevb.54.11169>
7. J. Perdew, K. Burke, M. Ernzerhof, Generalized gradient approximation made simple. Phys. Rev. Lett. **77**(18), 3865–3868 (1996). <https://doi.org/10.1103/PhysRevLett.77.3865>
8. G. Kresse, D. Joubert, From ultrasoft pseudopotentials to the projector augmented-wave method. Phys. Rev. B **59**(3), 1758–1775 (1999). <https://doi.org/10.1103/physrevb.59.1758>
9. P.E. Blöchl, Projector augmented-wave method. Phys. Rev. B **50**(24), 17953–17979 (1994). <https://doi.org/10.1103/physrevb.50.17953>
10. G. Fang, C. Liu, X. Wei, Q. Cai, C. Chen et al., Determining the preferable polarization loss for magnetoelectric microwave absorbers by strategy of controllably regulating defects. Chem. Eng. J. **463**, 142440 (2023). https://doi.org/10.1016/j.cej.2023.142440
11. L. Rao, Z. Liu, L. Wang, W. You, C. Yang et al., Dimensional engineering of hierarchical nanopagodas for customizing cross-scale magnetic coupling networks to enhance electromagnetic wave absorption. Adv. Funct. Mater. **33**(47), 2306984 (2023). <https://doi.org/10.1002/adfm.202306984>
12. B. Cai, L. Zhou, P.-Y. Zhao, H.-L. Peng, Z.-L. Hou et al., Interface-induced dual-pinning mechanism enhances low-frequency electromagnetic wave loss. Nat. Commun. **15**, 3299 (2024). <https://doi.org/10.1038/s41467-024-47537-5>
13. J.-C. Shu, M.-S. Cao, M. Zhang, X.-X. Wang, W.-Q. Cao et al., Molecular patching engineering to drive energy conversion as efficient and environment-friendly cell toward wireless power transmission. Adv. Funct. Mater. **30**(10), 1908299 (2020). <https://doi.org/10.1002/adfm.201908299>
14. R. Hu, J. Luo, H. Wen, C. Liu, J. Peng et al., Enhanced electromagnetic energy conversion in an entropy-driven dual-magnetic system for superior electromagnetic wave absorption. Adv. Funct. Mater. **35**(14), 2418304 (2025). <https://doi.org/10.1002/adfm.202418304>
15. N. Qu, H. Sun, Y. Sun, M. He, R. Xing et al., 2D/2D coupled MOF/Fe composite metamaterials enable robust ultra-broadband microwave absorption. Nat. Commun. **15**(1), 5642 (2024). <https://doi.org/10.1038/s41467-024-49762-4>
16. R. Tan, Y. Liu, W. Li, J. Zhou, P. Chen et al., Multi-scale dispersion engineering on biomass-derived materials for ultra-wideband and wide-angle microwave absorption. Small Meth. **8**(12), 2301772 (2024). <https://doi.org/10.1002/smtd.202301772>
17. R. Wang, Y. He, C. Tang, X. Wu, Q. Zhuang et al., Constructing crystalline/amorphous heterojunction in FeCo@C nanoboxes for enhanced electromagnetic wave absorption. Carbon **229**, 119494 (2024). <https://doi.org/10.1016/j.carbon.2024.119494>
18. Y. Zhou, C. Tang, S. Li, X. Wu, P. Zuo et al., Quantifying heterogeneous interface effect of Fe_3_O_4_(111)/C for enhanced low-frequency electromagnetic wave absorption. Carbon **232**, 119792 (2025). <https://doi.org/10.1016/j.carbon.2024.119792>
19. C. Ma, C. Zhang, M. Yuan, X. Guo, X. Liu et al., ZIF-67 derived CoNi@carbon/RGO composites with abundant heterogeneous interfaces for electromagnetic wave absorption. Appl. Surf. Sci. **665**, 160283 (2024). <https://doi.org/10.1016/j.apsusc.2024.160283>
20. M. He, X. Zhong, X. Lu, J. Hu, K. Ruan et al., Excellent low-frequency microwave absorption and high thermal conductivity in polydimethylsiloxane composites endowed by *Hydrangea*-like CoNi@BN heterostructure fillers. Adv. Mater. **36**(48), 2410186 (2024). <https://doi.org/10.1002/adma.202410186>
21. L. Li, Q. Ban, Y. Song, J. Liu, Y. Qin et al., Self-templating engineering of hollow N-doped carbon microspheres anchored with ternary FeCoNi alloys for low-frequency microwave absorption. Small **20**(50), 2406602 (2024). <https://doi.org/10.1002/smll.202406602>
22. L. Rao, L. Wang, C. Yang, R. Zhang, J. Zhang et al., Confined diffusion strategy for customizing magnetic coupling spaces to enhance low-frequency electromagnetic wave absorption. Adv. Funct. Mater. **33**(16), 2213258 (2023). <https://doi.org/10.1002/adfm.202213258>
23. M. He, J. Hu, H. Yan, X. Zhong, Y. Zhang et al., Shape anisotropic chain-like CoNi/polydimethylsiloxane composite films with excellent low-frequency microwave absorption and high thermal conductivity. Adv. Funct. Mater. **35**(18), 2316691 (2025). <https://doi.org/10.1002/adfm.202316691>
